# Whole Genome Sequencing and Comparative Genomics Analyses of *Pandoraea* sp. XY-2, a New Species Capable of Biodegrade Tetracycline

**DOI:** 10.3389/fmicb.2019.00033

**Published:** 2019-01-29

**Authors:** Xueling Wu, Xiaoyan Wu, Li Shen, Jiaokun Li, Runlan Yu, Yuandong Liu, Guanzhou Qiu, Weimin Zeng

**Affiliations:** ^1^School of Minerals Processing and Bioengineering, Central South University, Changsha, China; ^2^Key Laboratory of Biometallurgy, Ministry of Education, School of Minerals Processing and Bioengineering, Central South University, Changsha, China

**Keywords:** *Pandoraea* sp. XY-2, tetracycline biodegradation, genome sequencing, comparative genomic, tetracycline resistance

## Abstract

Few bacteria are resistant to tetracycline and can even biodegrade tetracycline in the environment. In this study, we isolated a bacterium *Pandoraea* sp. XY-2, which could biodegrade 74% tetracycline at pH 7.0 and 30°C within 6 days. Thereafter, we determined the whole genome sequence of *Pandoraea* sp. XY-2 genome is a single circular chromosome of 5.06 Mb in size. Genomic annotation showed that two AA6 family members-encoding genes and nine glutathione S-transferase (GSTs)-encoding genes could be relevant to tetracycline biodegradation. In addition, the average nucleotide identities (ANI) analysis between the genomes of *Pandoraea* sp. XY-2 and other *Pandoraea* spp. revealed that *Pandoraea* sp. XY-2 belongs to a new species. Moreover, comparative genome analysis of 36 *Pandoraea* strains identified the pan and specific genes, numerous single nucleotide polymorphisms (SNPs), insertions, and deletion variations (InDels) and different syntenial relationships in the genome of *Pandoraea* sp. XY-2. Finally, the evolution and the origin analysis of genes related to tetracycline resistance revealed that the six *tetA*(48) genes and two specificgenes *tetG* and *tetR* in *Pandoraea* sp. XY-2 were acquired by horizontal gene transfer (HGT) events from sources related to *Paraburkholderia, Burkholderia, Caballeronia, Salmonella, Vibrio, Proteobacteria, Pseudomonas, Acinetobacter, Flavimaricola*, and some unidentified sources. As a new species, *Pandoraea* sp. XY-2 will be an excellent resource for the bioremediation of tetracycline-contaminated environment.

## Introduction

Tetracycline represents a major proportion of the broad-spectrum antibiotics widely used in prevention and therapy of human and animal diseases and as promoters for animal growth (Huber, 1971; Mesgari Abbasi et al., 2011; Wan et al., 2013; Leong et al., 2016). Because antibiotics cannot be absorbed or metabolized completely by the animals, 30–90% of antibiotics are excreted into the environment via urine and feces as the unmetabolized parent compound (Bound and Voulvoulis, 2004). Residues of antibiotic has increased the frequency of horizontal gene transfer (HGT) and resistance gene fixation in genomes, leading to the development of diverse resistance genes in genomic islands (Gillings and Stokes, 2012). Residues of antibiotic in the environment could cause the emergence and development of antibiotic-resistant genes and bacteria, which brings profound negative impacts on the health of human and animals (Chee-Sanford et al., 2001; Daghrir and Drogui, 2013; Zhu et al., 2013). Compared with other conventional methods such as hydrolysis and photolysis, microbial degradation of tetracycline is considered to be an inexpensive, eco-friendly, and high efficient technology for the removal of tetracycline from the environment (Jiao et al., 2008; Migliore et al., 2012). In recent years, there are only a few tetracycline-degrading microorganisms that have been reported. Zhang et al. (2015) found that 57.8% tetracycline could be degraded by *Advenella kashmirensi* which was selected from sewage of a pharmaceutical factory. Leng et al. (2016) have isolated tetracycline-degrading bacteria *S. maltophilia* strain DT1 from sewage of a contaminated soil.

Most antibiotic-degrading bacteria are drug-resistance bacteria because they need to survive in the presence of antibiotics to play their role of degradation. There are three mechanisms of tetracycline resistance: a ribosomal protection mechanism, an efflux pump mechanism, and an enzymatic inactivation mechanism (Speer and Salyers, 1989; Thaker et al., 2010; Roberts et al., 2011). Leng et al. (2017) have found that ribosomal protection and efflux pump mechanism were presented in the tetracycline resistance strain *Stenotrophomonas maltophilia* DT1. Leski et al. (2013) found that various pathogens, such as *Klebsiella pneumoniae* and *Bacteroides fragilis*, contained a flavin-dependent monooxygenase encoded gene *tet*(X), which can inactivate tetracycline.

The third-generation sequencing platform PacBio RS II and annotation analysis of the whole genome data can offer increased read lengths, unbiased genome coverage and identify the genes involved in the xenobiotic biodegradation and drug-resistance (Ferrarini et al., 2013; Kim et al., 2015; Huang et al., 2018). Jung et al. (2012) have identified the genes related to pectin metabolism in pectin degrader *Alishewanella aestuarii* B11(T) based on genomic analysis. Moreover, comparative genomics analyses can reveal the molecular implications associated with genetic diversity affecting the evolutionary origin and how these genetic differences are generated among distinct lineages (Teng et al., 2016). In particular, the phylogeny, the genetic traits and the evolutionary history of specific resistance pathways can also be obtained through comparative genomic analysis (Wang et al., 2017). Comparative genomics analyses can shed light on the genetic basis underlying the degradation ability to organic contaminants of bacteria. Therefore, investigating the resistance mechanisms in the tetracycline resistant bacteria at the molecular level is important to predicting the environmental fate of antibiotics and assessing the biological impacts of antibiotics on the microbes in the environment.

The genus *Pandoraea* was first proposed in 2,000 following the isolation from the sputum of cystic fibrosis patients (Coenye et al., 2000). Today, a total of 10 species with valid nomenclature (Parte, 2014) in the genus *Pandoraea* have been described. Members of *Pandoraea* tend to exhibit broad resistance to ampicillin, extended-spectrum cephalosporins, aztreonam, aminoglycosides, and meropenem (Daneshvar et al., 2001; Stryjewski et al., 2003). To date, *Pandoraea* spp. have been considerably attractive for biotechnological applications with various degradation abilities such as lignin degradation, polychlorinated biphenyls (PCBs) biodegradation, and chlorobenzen degradation (Baptista et al., 2010; Dhindwal et al., 2011; Shi et al., 2013). In addition, *Pandoraea* spp. used for bioremediation were essentially isolated from the environment, such as rotten stumps, petroleum refinery waste, and municipal wastewater treatment plant (Sarkar et al., 2017; Fang et al., 2018; Yang et al., 2018). However, to our best knowledge, no documentation of tetracycline biodegradation in the genus of *Pandoraea* has ever been reported to date. Moreover, there are relatively few analyses on the comparative genomics of *Pandoraea* genus.

In the present study we report that a tetracycline-degrading bacterium, *Pandoraea* sp. XY-2, was isolated and its tetracycline biodegrading behavior under various time, temperature, and initial pH was investigated. To further investigate the genome structure and function of *Pandoraea* sp. XY-2, we performed whole genome sequencing and annotation on *Pandoraea* sp. XY-2 using the Pacific Biosciences RSII single-molecule real-time (SMRT) platform and other related functional annotation databases. We then calculated the average nucleotide identity (ANI) and digital DNA-DNA hybridization (dDDH) values between the available genomes of the whole *Pandoraea* group in order to ascertain whether *Pandoraea* sp. XY-2 represented a new species within the *Pandoraea* group. Additionally, we constructed the genomic evolution events involving this strain and compared the gene and genomic structure with those of closely related *Pandoraea* species to assess the phylogenetic relationship, genetic diversity and genomic structure variation. Finally, the genetic traits and evolutionary history of genes related to tetracycline resistance in genus *Pandoraea* were investigated. The insights gained in this study provides information useful for further clarifying the molecular mechanisms behind tetracycline biodegradation and resistance.

## Materials and Methods

### Chemicals, Sampling, and Media

All chemicals were purchased from Sinopharm Chemical Reagent Co. Ltd., including tetracycline standard, tryptone, yeast extract, NaCl, glucose, chromatography grade methanol and acetonitrile, analytical grade sodium dihydrogen phosphate, oxalic acid, disodium ethylenediaminetetraacetate, and citric acid.

The soil sample was obtained from the sludge in the sediment pool of a pig factory using tetracycline antibiotics as feed additive for a long-term (4 a) in Changsha. The average temperature and pH value of the sampling location in this study was 20°C and about 6.8. The latitude and longitude of our sampling locations are 28.1932 and 112.6973, respectively.

Lysogeny broth (LB) medium contained the following (in g L^−1^): tryptone, 10; yeast extract, 5; NaCl, 5; glucose, 10. The pH of LB medium was adjusted to 7.0–7.2 before autoclaving at 121°C for 20 min.

### Bacterial Strain Isolation and Identification

0.1 g soil sample was made into suspension with 10 mL sterile water. One milliliter supernatant was added to the LB medium (An additional volume of tetracycline stock solution was added to the culture medium so that the tetracycline concentration was 20 mg L^−1^). The mixed solution was incubated at 30°C on a dark shaker at 150 rpm. After 3 days, 0.5 mL of the solution was inoculated into the LB medium containing 40 mg L^−1^ tetracycline and the other conditions remained unchanged, and then cultured for 6 days. The above procedures were repeated until the final concentration of tetracycline reached 120 mg L^−1^. After the enrichment, the solution was serially diluted from 10^−1^ to 10^−9^, and then 0.5 mL of each diluted solution was added to petri dishes and daub uniformly. Six colonies with different morphology were obtained after multiple purifications, and then inoculated separately into LB medium for cultivation. Only the isolate with a high level of degradation efficiency of tetracycline was further analyzed. Finally, *Pandoraea* sp. XY-2 was identified through 16S rRNA sequence analysis as previously described (Mangwani et al., 2015).

### Analytical Methods of Residual Tetracycline

Concentration of tetracycline residues were analyzed by high-performance liquid chromatography (HPLC). A C18 reversed-phase column (Agilent Technologies) was operated at 30°C, with a 0.5 mL min^−1^ mobile phase consisting of 10 mmol L^−1^ sodium dihydrogen phosphate solution and acetonitrile. The injection volume was 20 μL, and the column gradient elution was monitored by a UV detector at 360 nm. Five milliliter solution from each reactor was centrifuged at 8,000 rpm at 4°C for 15 min. Four milliliter supernatant was added to a centrifuge tube containing 4 mL Na_2_EDTA-Mcllvaine buffer, then 1 mL n-hexane and 1 mL chloroform were added, and the mixture was shaked for 2 min and sonicated for 30 s. After the mixture was centrifuged at 8,000 rpm for 15 min, the supernatant was filtered through a 0.22 μm microporous membrane filter and preserved at 4°C for further testing with the previously described chromatographic conditions.

### Tetracycline Biodegradation Experiments

In order to obtain the optimal conditions for *Pandoraea* sp. XY-2 biodegrading tetracycline, we studied the growth of *Pandoraea* sp. XY-2 and the biodegradation percentage of tetracycline under various culture time (i.e., 1, 2, …, 8, 9 day), temperatures (i.e., 20, 25, 30, 35, 40°C) and initial pH values (i.e., 5.0, 6.0, 7.0, 8.0, 9.0). Only biodegradation was considered because hydrolysis of tetracycline could be ignored during the testing period (Supplementary Figure 1). In these experiments, the amount of each bottle of culture solution was 150 mL. *Pandoraea* sp. XY-2 culture solution was inoculated to LB medium containing 50 mg L^−1^ tetracycline at 3% inoculum concentration, and then placed on a dark shaker at 150 rpm. During the culture time tests, the temperature and initial pH were 30° C and 7.0, respectively. During the different temperature tests, the initial pH was 7.0. During the pH tests, the temperature was 30°C. The final growth of *Pandoraea* sp. XY-2 and the biodegradation percentage of tetracycline in temperature and pH tests were measured only at the 6th day. All experiments were performed in triplicates. The growth of *Pandoraea* sp. XY-2 was measured by a hemocytometer and the concentration of tetracycline in the medium was measured using HPLC.

### DNA Extraction, Genome Sequencing, Assembly, and Annotation

Genomic DNA of *Pandoraea* sp. XY-2 was extracted using the Genomic DNA Extraction Kit (Tian Gen biochemical technology Co. Ltd., Beijing, China).

Genomic DNA was sheared in a Covaris g-TUBE, and DNA fragments were then damage repaired, terminal repaired and purified using AMpure PB magnetic beads. SMRTbell libraries prepared with DNA Template Kit 2.0 and quantified by Qubit concentration. Agilent 2,100 was used to detect the size of the inserted fragments. Then, sequencing carried out using the PacBio RS II SMRT sequencing technology (Beijing Nuohe Zhiyuan Technology Co., Ltd., Beijing, China).

*De novo* genome assembly of PacBio reads for XY-2 (91,372 reads totaling 1,072,300,065 bp, mean length 11,735 bp, N50 length 16,735 bp) was conducted using the hierarchical genome assembly process (HGAP3 protocol and SMRT Link v.5.0.1 software) (Rhoads and Au, 2015). The mapping of assembled contigs onto long reads from the PacBio platform was performed by BlasR (Chaisson and Tesler, 2012). After mapping, long reads with high accuracy were assembled by the Celera Assembler v8.0 (Science New York, 2000). Then, the assembly resulted in one polished contig, which was a circular chromosome with no gaps.

Gene prediction carried out by GeneMarkS software (http://topaz.gatech.edu/). The tRNAscan-SE v1.3.1 and rRNAmmer v1.2 were used for identifying tRNA and rRNA, respectively (Schattner et al., 2005; Lagesen et al., 2007). Additionally, online Web-based tool Island Viewer v.3 was used for detecting genomic islands (Dhillon et al., 2015). To predict Interspersed Repeats, RepeatMasker was used (Chen, 2004). Tandem Repeats were identified for by using Tandem Repeat Finder v.4.09 (Benson, 1999). Finally, the gene functions were further investigated by BLASTP using the following five different function databases: the carbohydrate-active enzymes (CAZy) database (http://www.cazy.org/), the non-redundant (NR) protein database (http://www.ncbi.nlm.nih.gov/protein), the clusters of orthologous groups (COG) database (https://www.ncbi.nlm.nih.gov/COG/), the GO database (http://www.geneontology.org/), and the KEGG database (http://www.genome.jp/kegg/pathway.html).

### Average Nucleotide Identity (ANI) Calculations and Phylogenetic Analyses

Before ANI analysis, the 16S rRNA sequence of type strains of *Pandoraea* spp. were retrieved from the EzTaxon server (http://www.eztaxon.org/, Jongsik et al., 2007). Comparative sequence analysis of the 16S rRNA gene of *Pandoraea* sp. XY-2 agaist the type strains of *Pandoraea* spp. was implemented within the server. The phylogenetic tree of *Pandoraea* sp. XY-2 based on the 16SrRNA gene sequences was constructed using the NJ method in MEGA v7.0 with 1,000 bootstraps replications (Kumar et al., 2016).

Reference genomes for comparison purposes were retrieved from the GenBank database (http://www.ncbi.nlm.nih.gov/genbank/). Whole genome comparisons to determine the ANI between genome sequences were produced using JSpeciesws database (http://jspecies.ribohost.com/jspeciesws) that use Blast alignments to evaluate whole genome homologies. Cut-offs for species delineation were 95% ANI on 69% of conserved DNA according to Goris et al. (2007). In addition, we also calculate the dDDH values using Genome-to-Genome Distance Calculator (Alsaari et al., 2015). The 70% species cut-off of dDDH usually kept in taxonomic studies of bacteria (Konstantinidis and Tiedje, 2007).

The other 35 *Pandoraea* strains (Table 1) from NCBI database were selected to perform the phylogenetic analysis of *Pandoraea* sp. XY-2. Firstly, the single-copy core genes were identified by core-pan analysis between *Pandoraea* sp. XY-2 and the 35 *Pandoraea* strains. The number and total length of concatenated single copy genes were 1,903 and 27,797 characters. Then, the multiple sequences alignment of single-copy core genes was performed using MUSCLE software. Finally, phylogenetic tree was generated using the NJ method in Mega v7.0 with 1,000 bootstraps replications.

**Table 1:** Genomes statistical information of the 36 strains used in this study.

**Organism**	**GenBank accession**	**Level**	**Source**	**Size (Mb)**	**GC%**	**Gene**
*P. pnomenusa* DSM 16536^T^	CP009553	complete	clinical	5.39	64.9	4834
*P. pnomenusa* RB-44	CP006938	complete	environmental	5.39	64.9	4841
*P. pnomenusa* 3kgm	CP006900	complete	environmental	5.43	64.7	4886
*P. pnomenusa* RB38	CP007506	complete	environmental	5.38	64.8	4786
*P. pnomenusa* MCB032	CP015371	complete	environmental	5.82	64.6	5252
*P. pnomenusa* 6399	GCA_000807775	contig	clinical	5.58	62.8	4917
*P. pnomenusa* 7641	GCA_000807785	contig	clinical	5.57	62.8	4922
*P. pnomenusa* NCTC13160	GCA_900454355	contig	unkown	5.47	64.8	4959
*P. pnomenusa* RB44	GCA_000523065	contig	environmental	5.35	64.9	4820
*P. norimbergensis* DSM11628^T^	CP013480	complete	environmental	6.17	63.1	5439
*P. faecigallinarum* DSM23572^T^	CP011807	complete	environmental	5.24	63.7	4621
*P. thiooxydans* ATSB16^T^	CP014839	complete	environmental	4.46	63.2	4147
*P. thiooxydans* DSM25325^T^	CP011568	complete	environmental	4.46	63.2	4138
*P. oxalativorans* DSM23570^T^	CP011253	complete	environmental	5.63	63.1	4985
*P. apista* TF81F4	CP010518	complete	clinical	5.58	62.6	5005
*P. apista* TF80G25	CP011279	complete	clinical	5.61	62.6	5023
*P. apista* AU2161	CP011501	complete	clinical	5.57	62.6	4974
*P.apista* FDAARGOS_126	GCA_002951195	complete	clinical	5.33	62.7	4773
*P. apista* LMG16407^T^	GCA_001049515	contig	clinical	5.54	62.7	5015
*P. apista* Pa14367	GCA_003034885	contig	clinical	5.60	62.8	5060
*P. apista* Pa16226	GCA_002238015	contig	clinical	5.72	62.7	5185
*P. apista* DSM16535^T^	CP013481	complete	clinical	5.57	62.6	5043
*P. vervacti* NS15^T^	CP010897	complete	environmental	5.64	63.6	4893
*P. pulmonicola* DSM 16583^T^	CP010310	complete	clinical	5.87	64.3	5069
*P. pulmonicola* NCTC13159	GCA_900454575	contig	clinical	5.87	64.3	5085
*P. sputorum* DSM21091^T^	CP010431	complete	clinical	5.74	62.8	5071
*P. sputorum* NCTC13161^T^	LT906435	complete	clinical	5.74	62.8	5063
*Pandoraea* sp. 64-18	GCA_001899225	Scaffold	unkown	4.88	63.8	4393
*Pandoraea* sp. B-6	GCA_000282835	Scaffold	environmental	5.04	63.5	4626
*Pandoraea* sp. CB10b_02	GCA_002216225	contig	unkown	4.84	65.9	4638
*Pandoraea* sp. E26	GCA_000681415	contig	environmental	5.48	64.7	4904
*Pandoraea* sp. ISTKB	GCA_001721285	contig	environmental	6.37	63.2	5592
*Pandoraea* sp. PE-S2R-1	GCA_002179865	contig	environmental	6.23	63.1	5386
*Pandoraea* sp. PE-S2T-3	GCA_002179965	contig	environmental	6.18	63.2	5327
*Pandoraea* sp. SD6-2	GCA_000389825	Scaffold	environmental	5.77	62.5	5247
*Pandoraea* sp. XY-2^a^	CP030849	complete	environmental	5.06	63.7	4974

a *This study. The details source and references of all testing strains are shown in Supplementary Table S1*.

### Comparative Genomics Analyses

We used the Bacterial Pan Genome Analysis tool (BPGA) pipeline (Chaudhari et al., 2016) to identify orthologous groups among *Pandoraea* testing genomes and to extrapolate the pan-genome models of applying default parameters. The statistics information and details source of all testing strains used are listed in Table 1 and Supplementary Table S1. We defined the set of genes shared among all strains as their core genome, the set of genes shared with more than two but not all strains as their accessory genome, the set of genes not shared with other strains in each strain as the specific genes and the set of non-homologous genes with all testing genomes as the pan genome. Finally, functional categories were assigned to gene clusters by the KEGG annotation previously described.

The size of *Pandoraea* pan-genome was extrapolated implementing a power law regression function *P*_s_ = κn^γ^ using a built-in program of BPGA pipeline (Chaudhari et al., 2016), where *P*_s_ is the total number of non-orthologous gene families within its pan-genome, *n* is the number of tested strains, and both κ and γ are free parameters (Tettelin et al., 2008). The exponent γ < 0 suggests the pan-genome is “closed” considering the size of the pan-genome is reaching a constant as extra genomes adding in. Conversely, species is predicted to harbor an “open” pan-genome for 0 < γ < 1. Furthermore, the size of the core-genome was extrapolated fitting into an exponential decay function *F*_c_ = κ_c_exp^(−n/τc)^ + Ω, with a built-in program of BPGA pipeline (Chaudhari et al., 2016), where *F*_c_ is the number of core gene families, while κ_c_, τ_c_, and Ω are free parameters (Tettelin et al., 2005).

For variation analysis, we use *P. apista* DSM16535^T^ as reference strain. The single nucleotide polymorphisms (SNPs) were identified by aligning scaffolds to reference using MUMmer 3.06 package (Kurtz et al., 2004). Reliable SNPs were obtained after using BLAST, TRF, and Repeatmask software to predict the repeat region of the reference sequence, and filter out the SNPs located in the repeat region. InDels (insertion and deletion variations) were detected through gap extension alignment between the whole genome *de novo* assembly and the reference using LASTZ software (Harris, 2007). Synteny analysis was performed using MUMmer v3.06 package.

### Genes for Tetracycline Resistance

The coding sequences that are resistant to antibiotic was predicted using the Resistance Gene Identifier (RGI) software in the Comprehensive Antibiotic Resistance Database (CARD) (Mcarthur and Wright, 2015). After identifying the tetracycline resistance genes, each resistance gene was compared on NCBI database to find the genes with high similarity. Then, the evolutionary history of genes for tetracycline resistance was inferred using MEGA v7.0 (Kumar et al., 2016) with the Neighbor-Joining method with 1,000 bootstraps.

### GenBank Accession Numbers

The sequence data of the whole genome of *Pandoraea* sp. XY-2 have been deposited to NCBI data base, with the accession number of CP030849.

## Results

### Isolation and Identification of Tetracycline-Degrading Bacteria

After enrichment, isolation, and purification, 6 strains named XY-1, XY-2, XY-3, XY-4, XY-5, and XY-6 were obtained. The colony morphology of strain XY-1 was round, white, opaque, while that of strain XY-3 was dry, white, uneven edges. The colony morphology of strain XY-4, XY-5, and XY-6 was cotton-like, snowflake and fluffy shape, respectively. All the strains were capable of biodegrade tetracycline, and the biodegrade percentage were between 36 and 65%. The strain XY-2 was selected for further analyses due to its highest ability to biodegrade tetracycline. The colony of strain XY-2 on LB solid medium was smooth and moist, and was light milky white with neat edges. It is a gram-negative bacterium and has a short rod shape under a scanning electron microscope (Supplementary Figure 2) and has a size of ~0.7 μm × (1.2–3) μm. After the 16S rDNA sequence analysis of strain XY-2, it was concluded that strain XY-2 belongs to genus *Pandoraea* in molecular phylogenetic classification and it was named *Pandoraea* sp. XY-2.

### The Optimum of Biodegradation Parameters of Tetracycline by *Pandoraea* sp. XY-2

**Figure 1:**
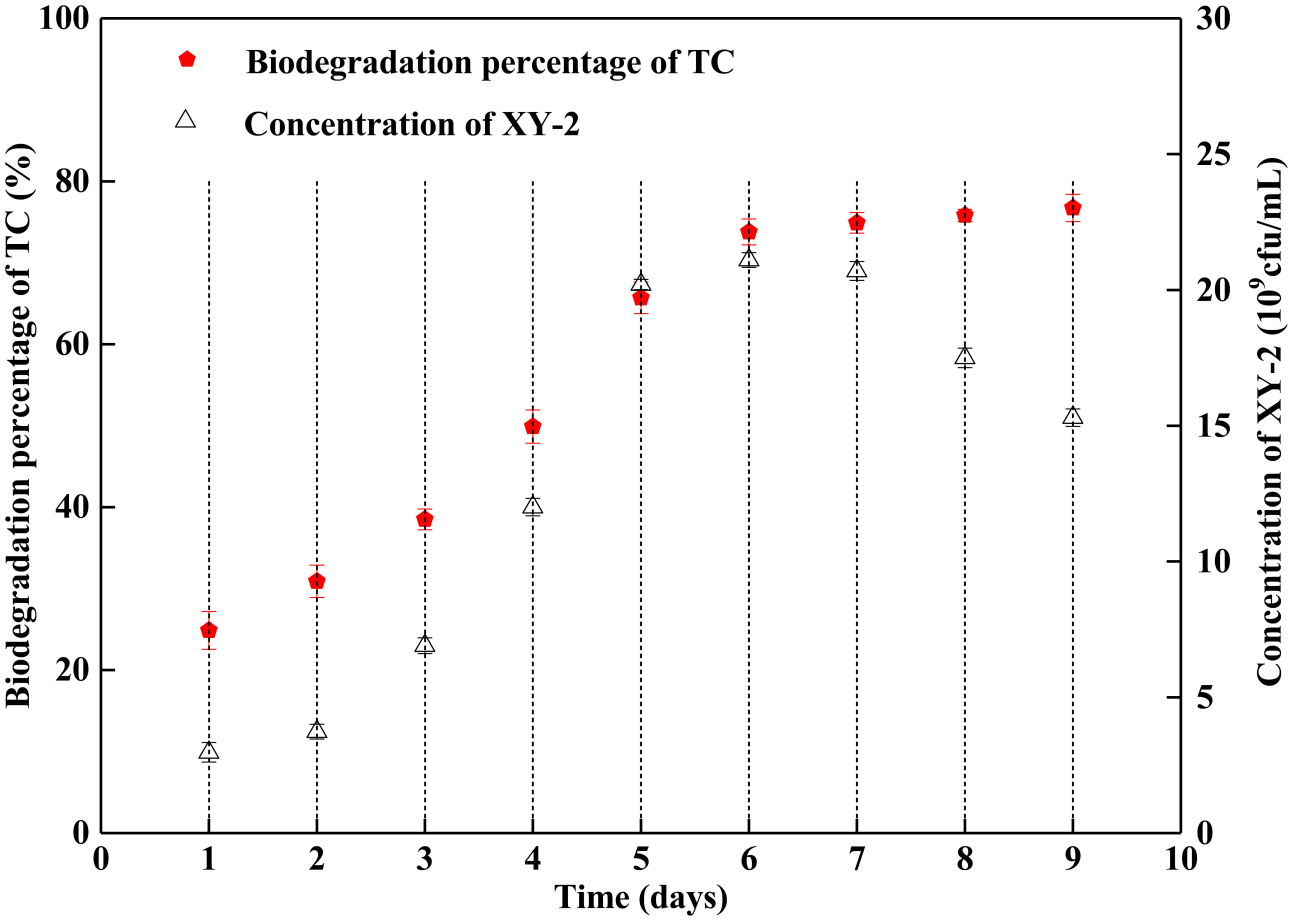
Effect of incubation time on the growth of *Pandoraea* sp. XY-2 and tetracycline biodegradation percentage. TC: tetracycline. XY-2: *Pandoraea* sp. XY-2. The initial concentration of tetracycline was 50 mg L^−1^. *Pandoraea* sp. XY-2 were cultured with an initial pH 7.0 at 30°C.

Figure 1 shows the effect of incubation time on the growth of *Pandoraea* sp. XY-2 and tetracycline biodegradation percentage. The growth of *Pandoraea* sp. XY-2 entered the logarithmic phase at the 3rd day, the biomass reached 2.1 × 10^10^ cfu/mL at the 6th day, and then the growth entered the stationary phase. The biodegradation percentage of tetracycline increased significantly during the first 6 days and it reached 73.8% at the 6th day. After that, there was almost no significant change with prolonged incubation.

**Figure 2:**
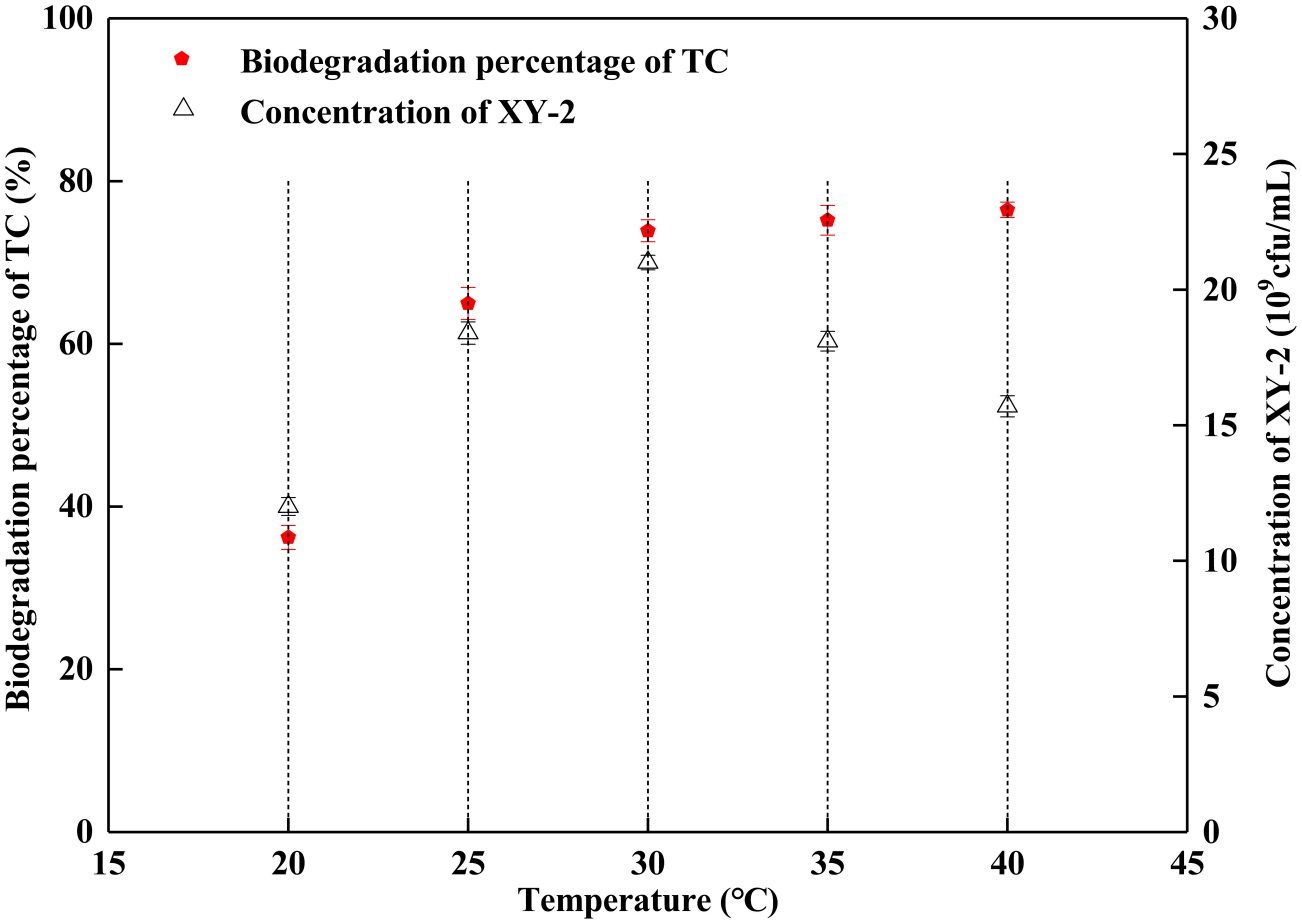
Effect of incubation temperature on the growth of *Pandoraea* sp. XY-2 and tetracycline biodegradation percentage. TC: tetracycline. XY-2: *Pandoraea* sp. XY-2. The effect of incubation temperature was tested at the 6th day. The initial concentration of tetracycline was 50 mg L^−1^. The initial pH was 7.0.

Effect of incubation temperature on the growth of *Pandoraea* sp. XY-2 and tetracycline biodegradation ability is shown in Figure 2. The growth of *Pandoraea* sp. XY-2 was greatly affected by temperature. With the increasing temperature from 30 to 40°C, the biomass of *Pandoraea* sp. XY-2 decreased to 1.57 × 10^10^ cfu/mL and the biodegradation percentage of tetracycline reached the maximum value and then remained stable at the 6th day.

**Figure 3:**
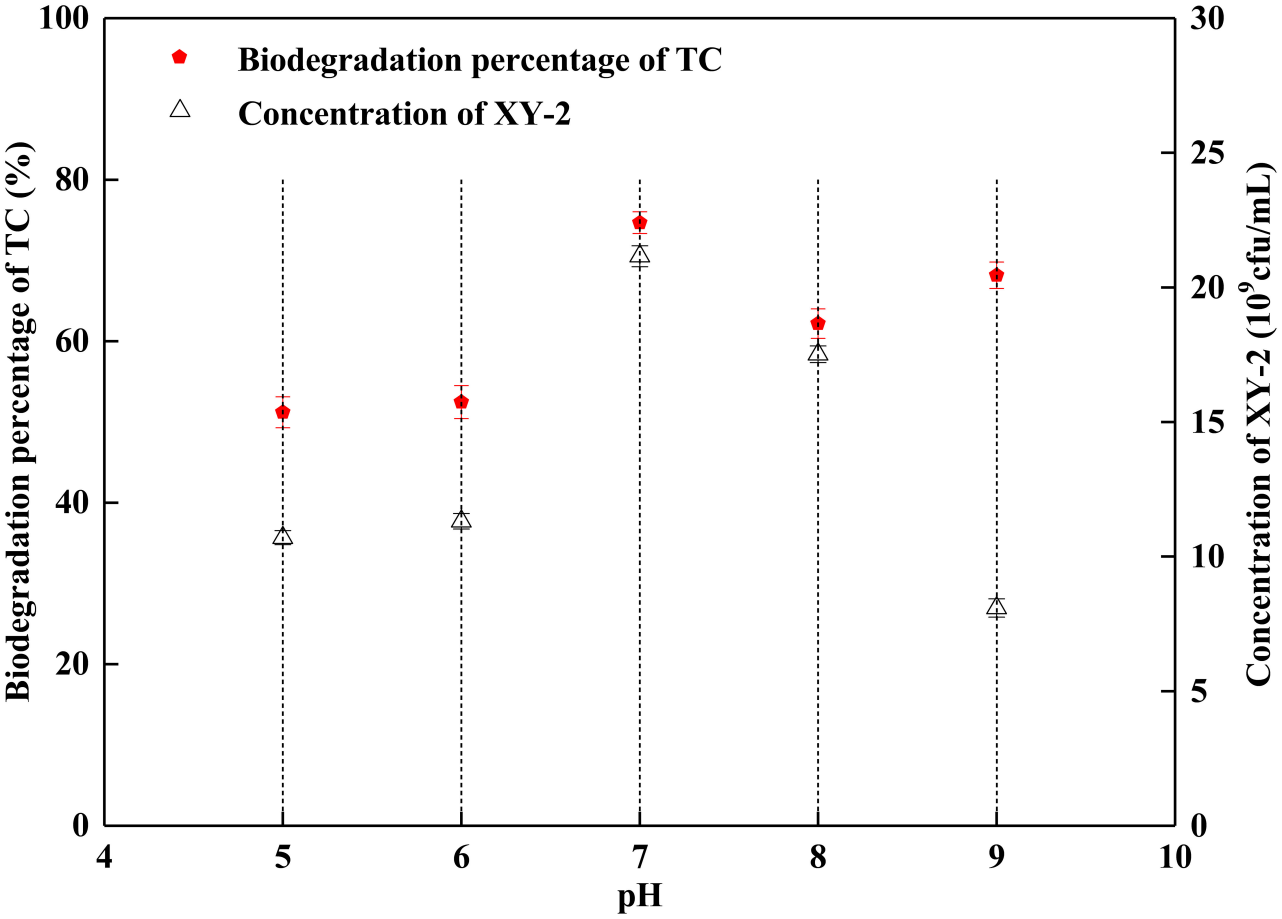
Effect of initial pH on the growth of *Pandoraea* sp. XY-2 and tetracycline biodegradation percentage. TC: tetracycline. XY-2: *Pandoraea* sp. XY-2. The effect of initial pH was tested at the 6th day. The initial concentration of tetracycline was 50 mg L^−1^. The temperature was 30°C.

The proper pH value can affect not only the growth rate of microorganisms, but also the activity of enzymes. In this study, the growth of *Pandoraea* sp. XY-2 was significantly inhibited when the initial pH was 5 and 9 (Figure 3). The *Pandoraea* sp. XY-2 grew most vigorously and the biodegradation percentage of tetracycline reached the maximum value at pH 7. It was concluded that the growth of *Pandoraea* sp. XY-2 and tetracycline biodegradation percentage generally showed a trend of increasing first and decreasing then with the increasing initial pH.

Combined with the growth of *Pandoraea* sp. XY-2 and tetracycline biodegradation percentage, we selected a pH of 7, temperature of 30°C and culture period of 6 day as the optimum culture conditions for subsequent experiments.

### Genome Characteristics and Functional Annotation of *Pandoraea* sp. XY-2

**Figure 4:**
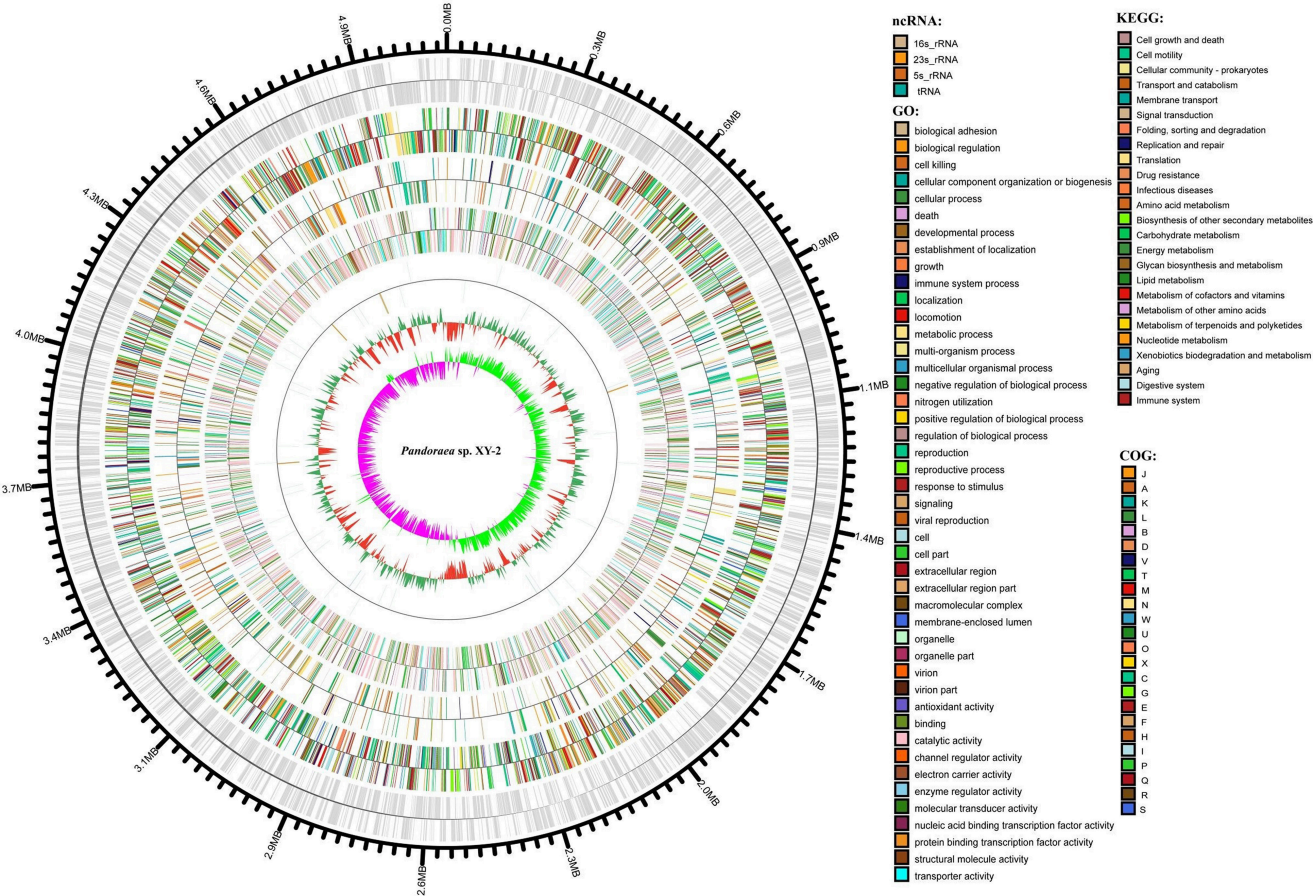
Circular representation of *Pandoraea* sp. XY-2. Based on, tRNA and rRNA, GO, KEGG, COG results, circular diagrams of chromosomes are drawn. The concentric circles show (reading outwards): GC skew, GC content, tRNA and rRNA, GO, KEGG, COG, genes, DNA coordinates.

After sequencing, a total of 91,372 reads were obtained. Genome characteristics of the newly sequenced genome of *Pandoraea* sp. XY-2 are shown in Table 2. The *Pandoraea* sp. XY-2 has a single circular chromosome and the estimated genome size was 5,056,206 bp with a GC content of 63.76% (Figure 4). No plasmid was found in the genome sequence of this bacterium. The number of predicted genes was 4,974 with an estimated total length of 4,382,283 bp which makes up 86.67% of the entire genome. Out of all the predicted genes, 3,489 (70%) could be functionally annotated in the NCBI non-redundant (nr) database. A total of 1,485 hypothetical proteins were predicted for XY-2. There are 77 RNA genes consisting of 65 tRNA and 12 rRNA (4 5S rRNA, 4 16S rRNA, and 4 23S rRNA) genes. Eight GIs were detected in the genome of the *Pandoraea* sp. XY-2. Approximately 5,470 bp of interspersed repeats were found in the genome of the *Pandoraea* sp. XY-2, which accounted for 0.1082% of its genome size, whereas 27,270 bp of tandem repeats were found, which accounted for 0.5393% of its genome size. Tandem repeats were the predominant type of repeat region, accounting for 83.3% of the repeat regions in the *Pandoraea* sp. XY-2 genome.

**Table 2:** Genome characteristics of the newly sequenced genome of *Pandoraea* sp. XY-2.

**Issue**	**Number**
Genome size (bp):	5,056,206
GC content (%):	63.76
Gene number:	4,974
Gene length (bp):	4,382,283
Gene average length (bp):	881
Gene length/genome (%):	86.67
GC content in gene region (%):	64.3
tRNA number:	65
rRNA number:	12
GIs number:	8
Interspersed repeat number:	62
Total length (bp):	5,470
In genome (%):	0.1082
Tandem repeat number:	508
Total length (bp):	27,270
In genome (%):	0.5393

Secreted CAZymes are crucial for bacteria biological activity. CAZymes are divided into five classes: glycoside hydrolases (GHs), carbohydrate esterases (CEs), polysaccharide lyases (PLs), glycosyltransferases (GTs), and auxiliary activities (AAs). Based on the CAZy annotation pipeline, we identified 65 CAZymes, including 5 AAs, 16 GHs, 30 GTs, 3 CEs, and 11 carbohydrate binding modules (CBMs) in the *Pandoraea* sp. XY-2 genome (Supplementary Figure 3). In the present study, CAZyme annotation revealed that *Pandoraea* sp. XY-2 contains a total of 2 families with 5 AAs in its genome sequence. Additionally, AA family classification also revealed that the components of AAs were 3 AA3 family members [glucose-methanol-choline (GMC) oxidoreductase, alcohol oxidase, aryl-alcohol oxidase/glucose oxidase, cellobiose dehydrogenase, pyranose oxidase), and 2 AA6 family members (1,4-benzoquinone reductase).

Clusters of orthologous groups (COG) database annotation showed that a number of genes are related to the basic functions of bacterial cells, including amino acid transport and metabolism (E), transcription (K), and energy production and conversion (C) (Supplementary Figure 4). Particularly, the number of genes participated in carbohydrate transport and metabolism (G), secondary metabolites biosynthesis, transport and catabolism (Q) and intracellular trafficking, secretion, and vesicular transport (U) were 244 (5.4%), 166 (3.6%), and 79 (1.7%), respectively. The GO annotation revealed that cellular processes and metabolic processes are very active in biological process; cell and cell part play a dominant role in cellular component; binding and catalytic activity play important roles in molecular function (Supplementary Figure 5). Furthermore, KEGG annotation showed that a total of 115 genes participated in the xenobiotic biodegradation and metabolism (Supplementary Figure 6 and Supplementary Table S2). It is worth noting that there were 5 genes encoding monooxygenase, 9 genes encoding dioxygenase, nine genes encoding glutathione S-transferase (GSTs) and 92 genes encoding other types of enzymes. Previous reports showed that tetracycline can be more readily biodegradable by using the detoxifying enzyme GSTs (Park and Choung, 2007). Accordingly, we selected all the 9 GSTs-encoding genes in *Pandoraea* sp. XY-2 together with GSTs-encoding genes from close-related strains for comparative analysis to shed light on the variation in the ability to degrade tetracycline between *Pandoraea* spp. The phylogenetic analysis of nine GSTs-encoding genes derived from *Pandoraea* sp. XY-2 and other close-related strains revealed that the GSTs-encoding genes were present in both environmental and clinical isolates and the evolution of these genes had no major variation (Supplementary Figure 7). We also calculated the GSTs-encoding genes contents in *Pandoraea* spp. and the results showed that the contents of these genes did not change significantly between environmental and clinical isolates (Supplementary Table S1).

### ANI Calculations and Phylogenetic Analyses

The phylogenetic tree of *Pandoraea* sp. XY-2 based on 16SrRNA gene sequence was shown in Supplementary Figure 8. *Pandoraea* sp. XY-2 was most closely related to the *P. apista* LMG16407^T^ (99.59 similarity). The 16S rRNA gene of *Pandoraea* sp. XY-2 having a similarity of 96.9–99.72% compared to the type strains of *Pandoraea* spp. (Supplementary Table S3). (Yarza et al., 2008) have reported that a new proposed genus would have ~6% divergence in 16S rRNA gene sequence from its closest genus. Therefore, *Pandoraea* sp. XY-2 was not a new genus. Stackebrandt and Ebers (2006) suggested that a 16S rRNA gene sequence similarity of 98.7–99% should become the boundary for delineation of prokaryotic species. Above this value, there is a need to prove by genome to genome comparisons whether distinct isolates belong to the same species. Accordingly, genomes comparisons of *Pandoraea* sp. XY-2 against all *Pandoraea* spp. were carry out for species circumscription.

ANI is considered to be the most relevant comparative parameter used for bacterial species delineation (Rosselló-Móra and Amann, 2015). Using ANI, the bacterial species delimitation borders can be set to about 94–96% identity, which would generally represent ~70% dDDH (Richter and Rossellómóra, 2009). Table 3 contain the matrix of nucleotide identities between whole genomes calculated under JSpeciesws database. The ANI% values of *Pandoraea* sp. XY-2 against all reference strains were < 85% (Table 3), which was a value far below the threshold of 94–96% identity that would serve as a boundary for species circumscription. Besides, the dDDH results showed that the dDDH% values of *Pandoraea* sp. XY-2 against all reference strains ranging from 14.9 to 52.2% (Supplementary Table S4).

**Table 3:** Average nucleotide identities (ANI) analysis. Genome comparison of *Pandoraea* sp. XY-2 and other *Pandoraea* species.

	**XY.2**	**DSM21091^**T**^**	**NS15^**T**^**	**TF81F4**	**TF80G25**	**LMG16407^**T**^**	**AU2161**	**DSM23572^**T**^**	**DSM23570^**T**^**	**MCB032**	**3 kgm**	**RB-44**	**DSM16583^**T**^**	**ISTKB**	**SD6-2**
*Pandoraea* sp. XY-2	*	82.7	82.8	84.2	84.2	84.2	84.2	82.9	83.0	83.1	83.1	83.1	83.2	83.3	84.5
*P. sputorum* DSM21091^T^	82.7	*	84.2	83.1	83.1	83.1	83.1	84.6	92.3	82.7	82.6	82.7	82.7	84.9	82.9
*P. vervacti* NS15^T^	82.8	84.2	*	83.0	83.0	83.0	83.0	85.6	84.1	82.8	82.8	82.8	82.8	85.0	82.9
*P. apista* TF81F4	84.1	83.0	82.9	*	100.0	99.1	100.0	83.1	83.1	83.0	83.0	83.1	83.1	83.4	87.5
*P. apista* TF80G25	84.1	83.0	82.9	100.0	*	99.1	100.0	83.1	83.1	83.0	83.0	83.1	83.1	83.4	87.5
*P. apista* LMG16407^T^	84.1	83.0	82.9	99.1	99.1	*	99.1	83.0	83.2	83.0	83.0	83.0	83.1	83.4	87.5
*P. apista* AU2161	84.2	83.1	83.1	99.9	99.9	99.1	*	83.1	83.2	83.1	83.2	83.2	83.2	83.4	87.5
*P. faecigallinarum* DSM23572^T^	83.0	84.6	85.8	83.2	83.2	83.1	83.2	*	84.6	82.8	82.9	82.9	83.1	85.5	83.1
*P. oxalativorans* DSM23570^T^	82.9	92.3	84.1	83.0	83.0	83.0	83.0	84.1	*	82.6	82.6	82.5	82.7	84.9	82.8
*P. pnomenusa* MCB032	83.1	82.8	82.8	83.1	83.1	83.0	83.1	82.8	82.8	*	99.0	98.8	83.2	83.2	83.1
*P. pnomenusa* 3 kgm	83.1	82.8	82.9	83.2	83.2	83.2	83.3	83.0	83.0	99.0	*	99.3	83.5	83.3	83.2
*P. pnomenusa* RB-44	83.2	82.8	82.9	83.2	83.2	83.2	83.2	83.0	83.0	99.1	99.4	*	83.5	83.4	83.2
*P. pulmonicola* DSM16583^T^	83.2	82.7	82.9	83.2	83.2	83.3	83.2	83.1	83.1	83.4	83.4	83.4	*	83.2	83.3
*Pandoraea* sp. ISTKB	82.7	84.6	84.5	83.1	83.1	83.1	83.1	85.0	84.6	82.7	82.7	82.7	82.7	*	83.1
*Pandoraea* sp. SD6-2	84.3	82.8	82.8	87.4	87.4	87.4	87.4	82.9	83.0	83.0	82.9	83.0	83.2	83.2	*

*Figures in red denote strains belonging to the same species. The asterisk indicates that the strain is compared to itself, which does not provide valuable information*.

**Figure 5:**
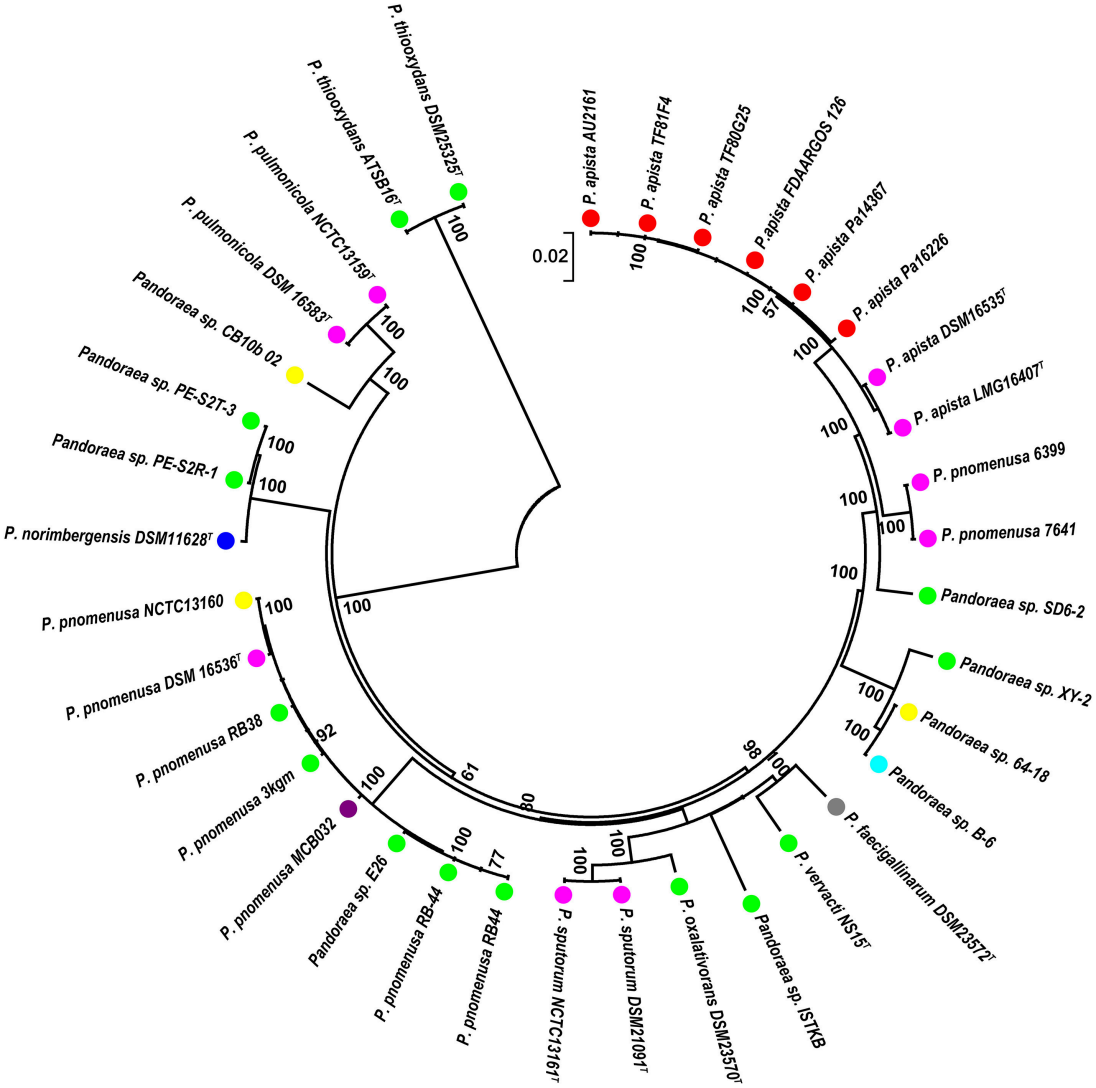
Phylogenetic analysis of *Pandoraea* sp. XY-2 based on all of the single-copy core genes among 36 whole genome sequences. The phylogeny tree was generated using the NJ method in Mega 7.0 with 1,000 bootstraps replications. The number and total length of concatenated single copy genes were 1,903 and 27,797 characters. The whole genome sequences of 35 other *Pandoraea* strains were retrieved from GenBank. The source of each strain was marked in different colors. Red, sputum; Pink, CF patient; Green, soil; Yellow, unknown; Blue, bamboo slips; Gray, oxalate-enriched culture; Purple, bioreactor; Navy blue, food.

A phylogenetic analysis based on the single-copy core genes was conducted to determine the relationship of *Pandoraea* sp. XY-2 with other 35 *Pandoraea* strains. Except for the sequencing data of *Pandoraea* sp. XY-2, other whole genomic data of 35 *Pandoraea* strains were downloaded from NCBI. The *Pandoraea* sp. XY-2 was clustered together with *Pandoraea* sp. 64–18 and *Pandoraea* sp. B-6 (Figure 5), which was indicative of intimate intragenus relationship among *Pandoraea* sp. XY-2, *Pandoraea* sp. 64–18 and *Pandoraea* sp. B-6. In addition, *Pandoraea* sp. XY-2 branches near the base of the *Pandoraea* sp. 64–18 and *Pandoraea* sp. B-6. We inferred that *Pandoraea* sp. XY-2 was likely arised before the *Pandoraea* sp. 64–18 and *Pandoraea* sp. B-6.

### Comparative Genomics Analyses

**Figure 6:**
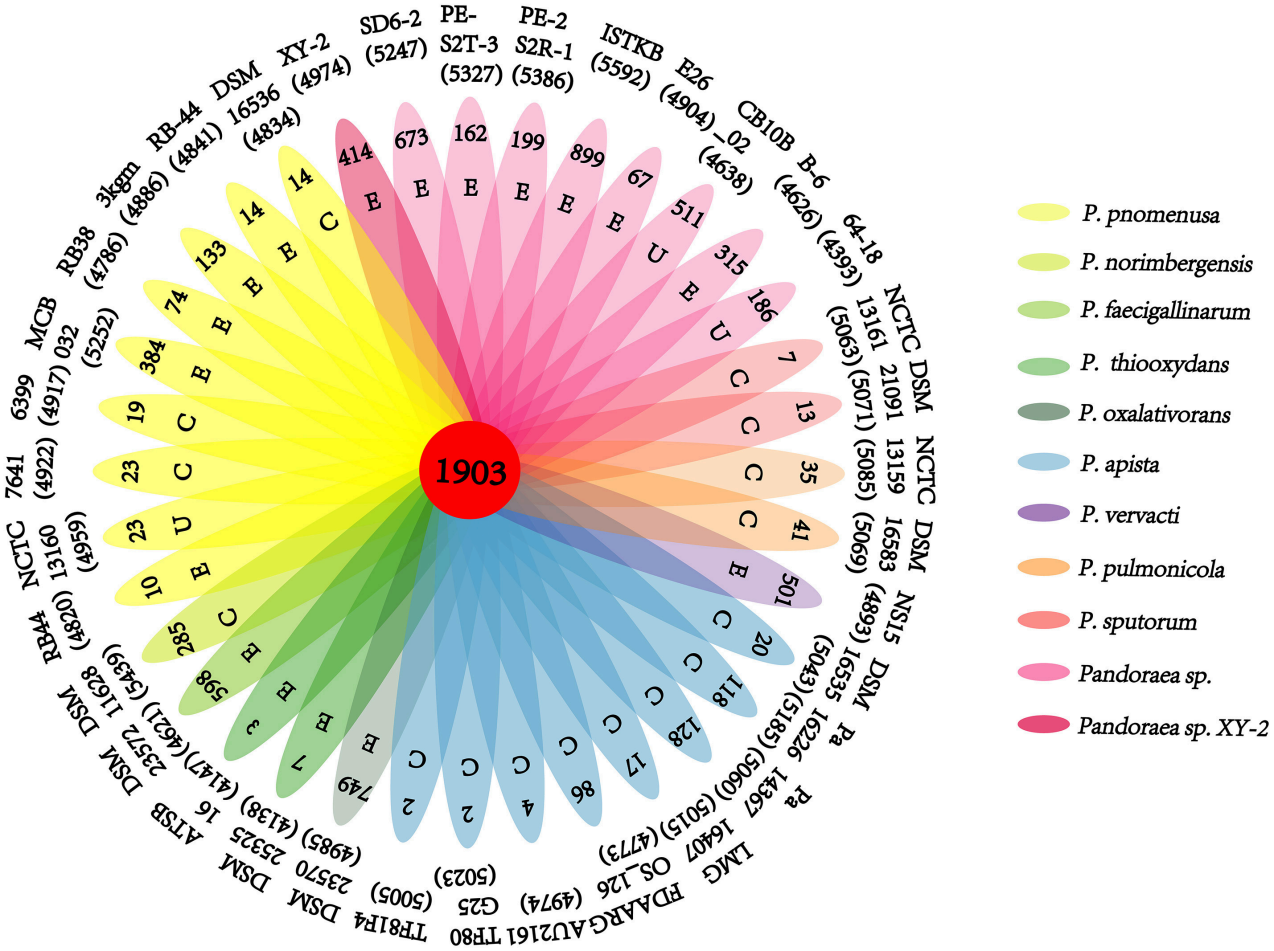
Pan-genome analysis of strains in the genus *Pandoraea*. Petal diagram of Pan-genome. The total number of protein coding genes within each genome is listed below the strain name. Numbers in non-overlapping portions of each oval show the number of CDSs specific to each strain. Letters in non-overlapping portions of each oval show the source of each strain (E, environment; C, clinical; U, unknown). The center is the number of orthologous coding sequences shared by all strains (i.e., the core genome).

A pan-genome for the *Pandoraea* sp. XY-2 and 35 other *Pandoraea* strains was determined by PGAP pipeline. The total pan-genome for the 36 compared *Pandoraea* strains encompasses 186,995 putative protein-coding genes. Of these, 1,903 (1.02% of total pan-genome) were core conserved genes across all 36 strain genomes compared. Out of these 186,995 genes, 2,280 (1.22%) were represented in the accessory genomes of *Pandoraea* sp. XY-2. After comparing strain-specific genes, the *Pandoraea* sp. ISTKB had the highest amount of these (*n* = 899), while the isolate sequenced in this study had 414 specific genes. The number of specific genes in all 36 strains ranges from 2 to 899 (Figure 6). The KEGG annotation of specific genes of *Pandoraea* sp. XY-2 showed that a total of 10 genes involved in the environmental information processing, including one TetR family protein-encoding gene *tetR* (XY.2_GM004468) and one DHA1 family protein-encoding gene *tetG* (XY.2_GM004469) (Supplementary Figure 9).

**Figure 7:**
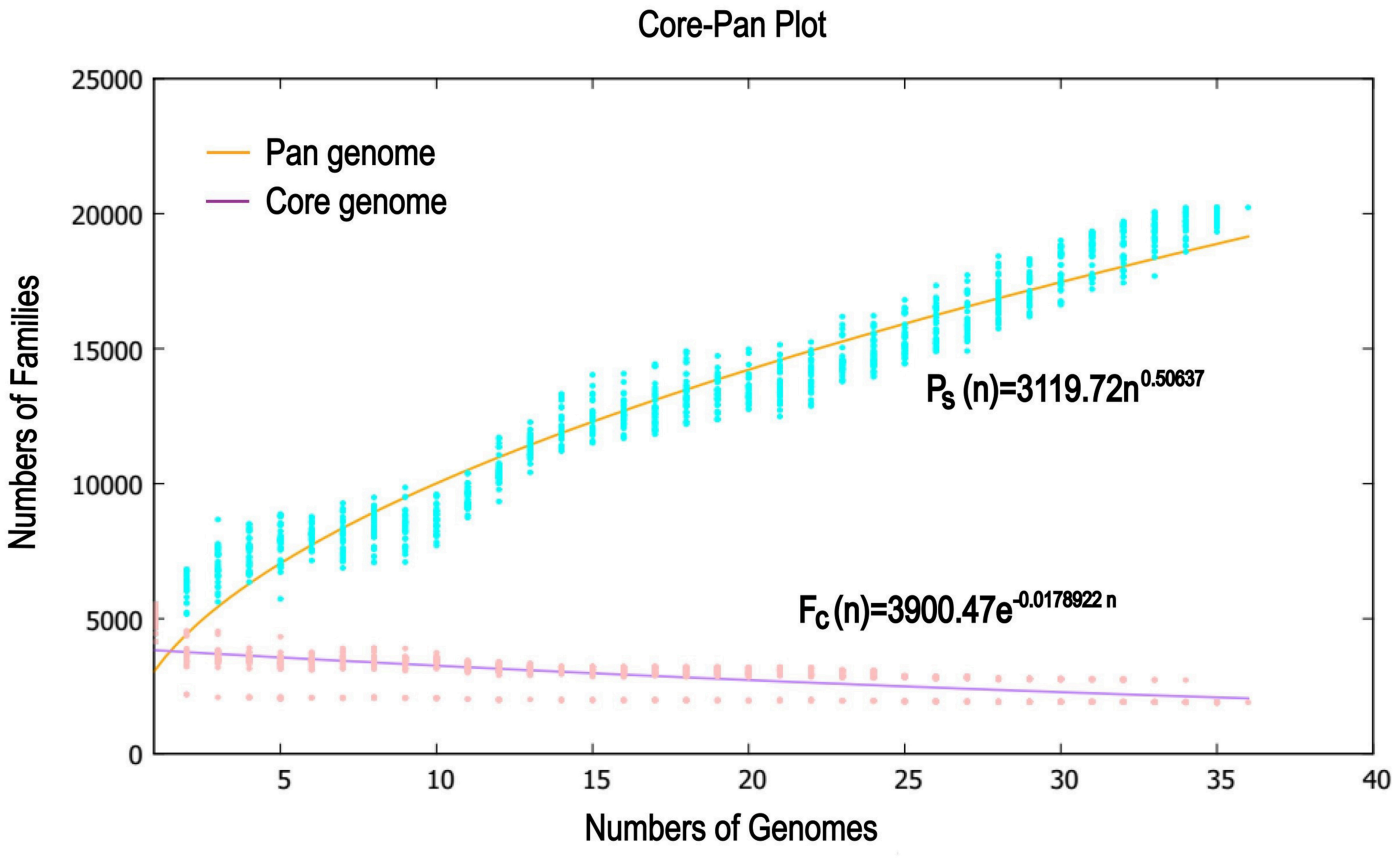
Mathematical modeling of pan genome and core genome of 36 *Pandoraea* strains.

Previous reports showed that mathematical extrapolation of pan-genome would be highly reliable provided that sufficient genomes (>5) are involved (Vernikos et al., 2015). The deduced power law regression function [*P*_s_ (*n*) = 3119.72 *n*^0.50637^] revealed that the pan-genome of *Pandoraea* had a parameter (γ) of 0.50637 falling into the range 0 < γ < 1, which indicating that the pan-genome was open (Figure 7) (Tettelin et al., 2008). In addition, the deduced exponential regression [*F*_c_ (*n*) = 3900.47e^−0.0178922n^] revealed that the extrapolated curve of core genome followed a steep slope, reaching a minimum of 1,903 gene families after the 36th genome was added (Figure 6).

The other 18 *Pandoraea* strains, which genome sequences level were complete, from NCBI database were selected to perform the phylogenetic analysis of *Pandoraea* sp. XY-2. The results showed that *Pandoraea* sp. XY-2 was clustered together with *P. apista* DSM16535^T^ (Supplementary Figure 10). Combined with that it has not yet been reported that tetracycline could be biodegraded by *Pandoraea* spp., so we selected *Pandoraea* sp. XY-2 and *P. apista* DSM16535^T^ to perform a genome-wide comparison. The genome-wide comparison identified all the SNPs, a total of 372,574 high-quality SNPs for *Pandoraea* sp. XY-2 were obtained (Supplementary Table S5). Most of the high-quality SNPs in the *Pandoraea* sp. XY-2 were located in CDS regions (333,149, 89.42%), with only 39,425 (10.58%) located in intergenic regions. A total of 67,476 non-synonymous SNPs and 264,974 synonymous SNPs were located within CDS regions, which resulted in a non-synonymous/synonymous (dN/dS) ratio of 0.255.

**Figure 8:**
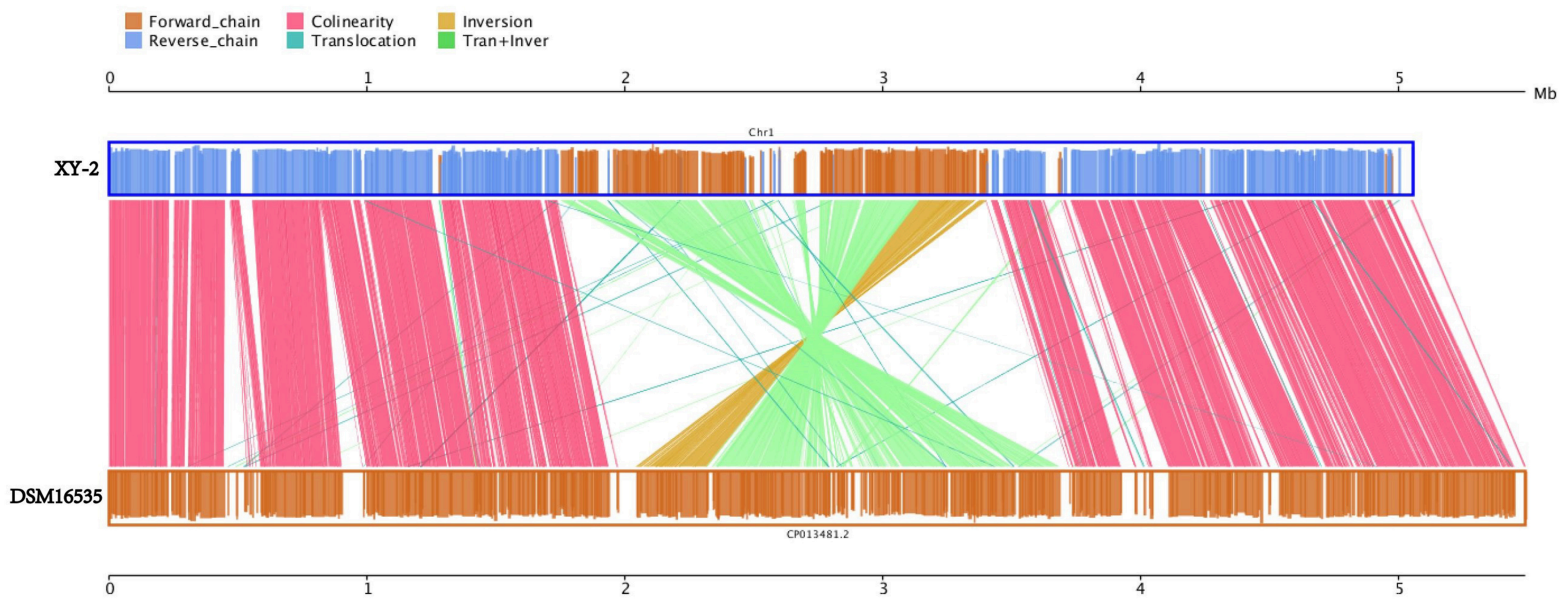
Synteny comparison of genomes between *Pandoraea* sp. XY-2 and *P. apista* DSM16535^T^. In the upper and lower axes, the brown frame represents the genome forward chain, the blue frame represents the genome reverse chain, the height of the color filled in the frame represents the similarity degree of the comparison. The color comparison type of the linked graph between the upper and lower axes: Collinear (red), Translocation (green), Inversion (yellow), and Tran+Inver (light green).

Gene gains and losses have been considered as one of the most significant contributors to functional changes (Nei and Rooney, 2005). We analyzed the insertion/deletion variations (InDels) between *Pandoraea* sp. XY-2 and *P. apista* DSM16535^T^ and the results are shown in Supplementary Table S6. We identified 581 InDels in the chromosome of *Pandoraea* sp. XY-2 and the proportion of base deletion (60.76%) is higher than that of base insertion (39.24%). Most of the InDels were located in intergenic regions (388, 66.78%). Furthermore, synteny comparison of genomes between *Pandoraea* sp. XY-2 and *P. apista* DSM16535^T^ showed that the two genomes have 550 collinear blocks covering 73.91 % of *Pandoraea* sp. XY-2 genome. On the other hand, some regions between *Pandoraea* sp. XY-2 and *P. apista* DSM16535^T^ displays inversion, translocation, and Tran+Inver, revealing different syntenial relationships to the reference sequence (Figure 8).

### The Origin and Evolution of Tetracycline Resistance Genes

From CARD analysis, a total of 218 genes were identified as having a role in the resistance to various antibiotics, of which 9 were tetracycline resistance genes (Supplementary Table S7). The type and number of tetracycline resistance genes were 6 *tetA*(48), 1 *tetB*(P), 1 *tetG*, and 1 *tetT* (Supplementary Table S8). Resistance is primarily due to either energy-dependent efflux of tetracycline or protection of the ribosomes from the action of tetracycline. *TetA*(48) and *tetG* belong to the efflux pump gene, while *tetB*(P) and *tetT* belong to the ribosome protection gene (Roberts, 1996). Previous studies have shown that efflux pumps play a major role in both organic contaminants tolerance and bioremediation (Fernandes et al., 2003), and combined with KEGG annotation results of specific genes in *Pandoraea* sp. XY-2, so we selected *tetA*(48), *tetG*, and *tetR* genes for further origin and evolution analysis.

**Figure 9:**
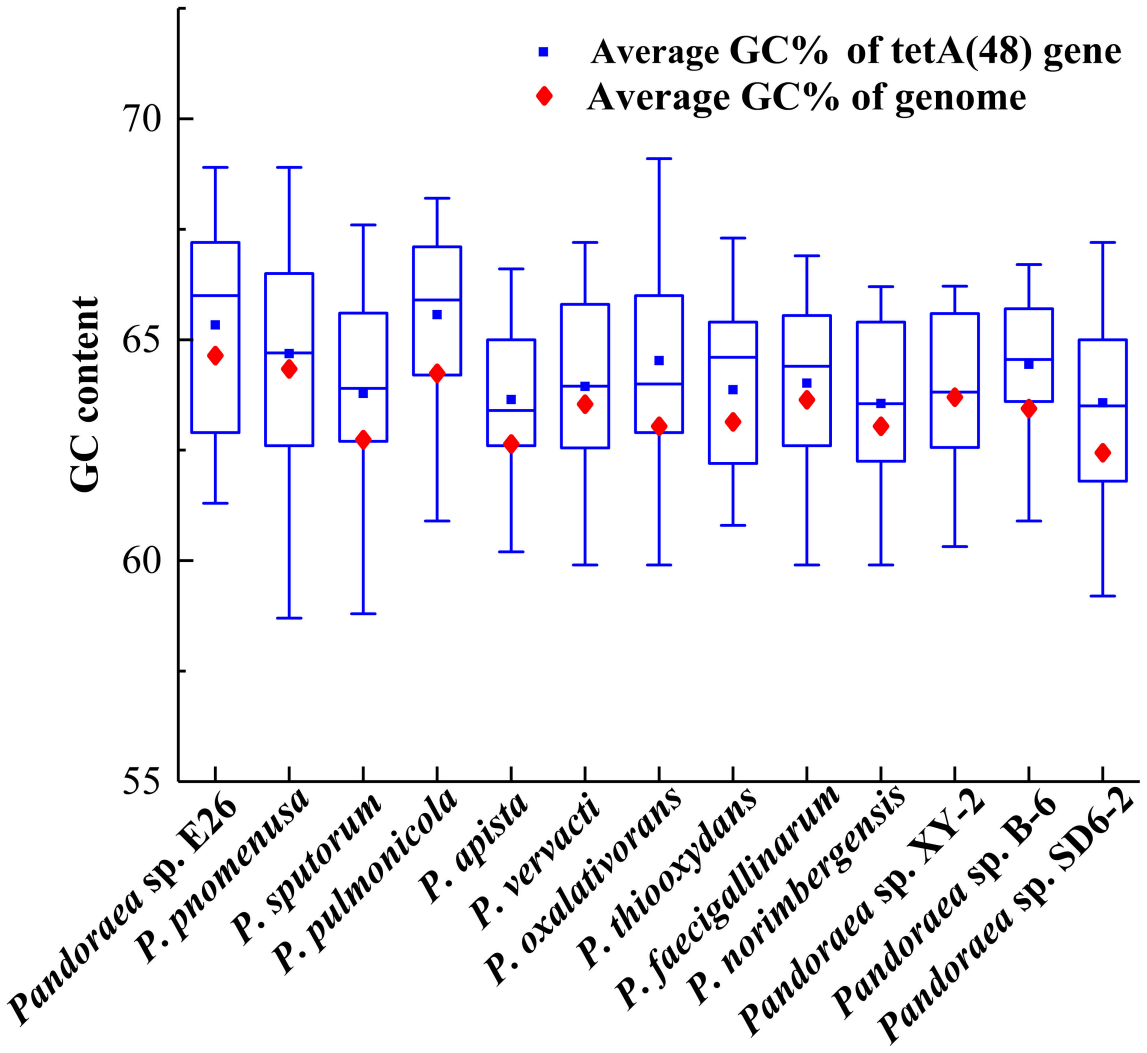
Comparison of GC contents of the *tetA*(48) genes with those of the average of the entire genomes.

The deviant GC content is used as a detect method of HGT (Xie et al., 2014). The average GC contents of the *tetA*(48) genes were higher than those of the average of the entire genomes (63.33–65.57 vs. 62.5–64.7) except that the average GC content of the *tetA*(48) genes in *Pandoraea* sp. XY-2 was slightly lower than that of the average of the genome (63.72 vs. 63.76) (Figure 9). It is worth noting that the *tetG* gene and *tetR* gene were specific genes of Pandoraea sp. XY-2, whose GC content were significantly lower than that of the genome (57.74 vs. 63.76 and 59.17 vs. 63.76, respectively). These data showing variation of GC content between tetracycline resistance genes and the corresponding genomes, which indicated that these tetracycline resistance genes may be acquired in *Pandoraea* strains by HGT.

**Figure 10:**
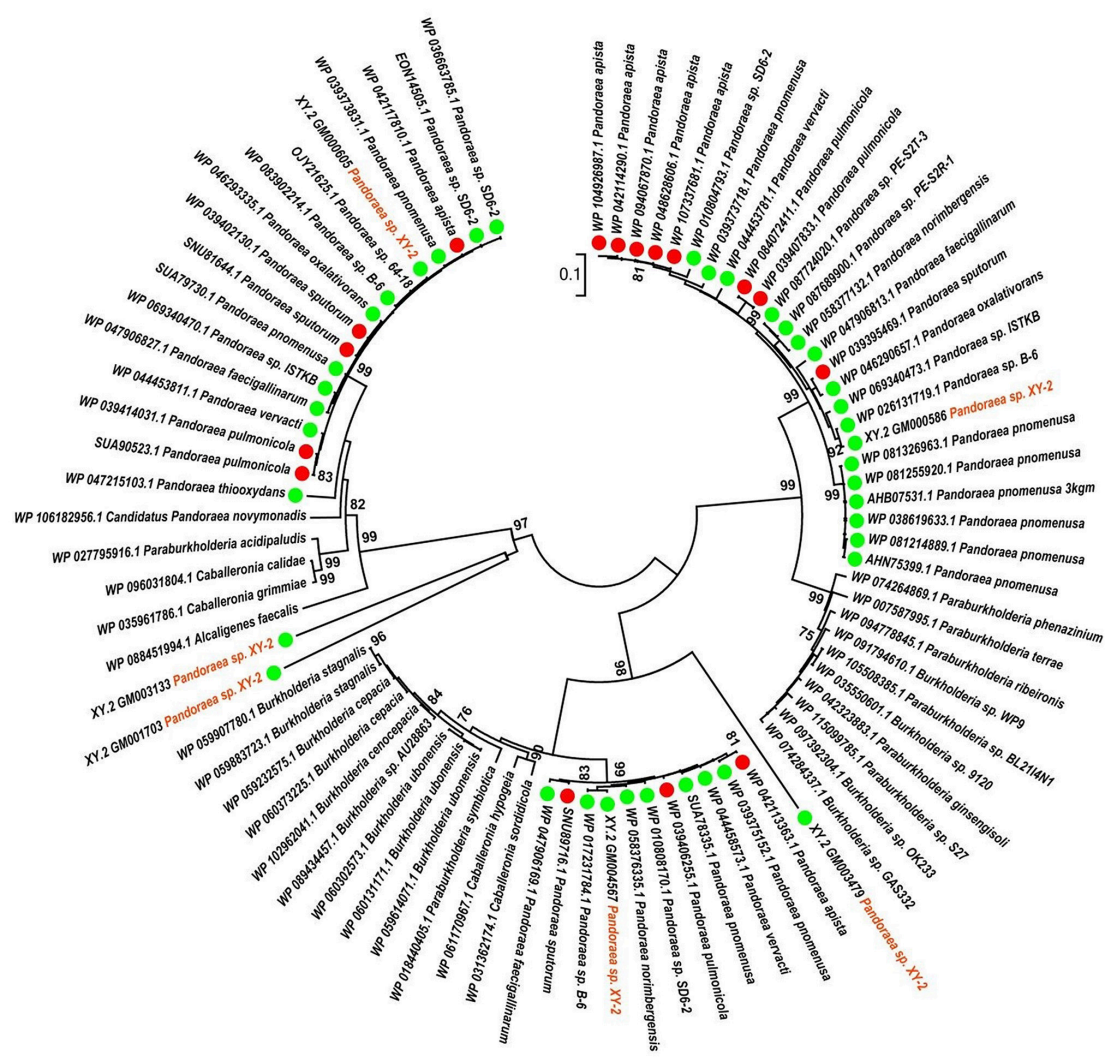
Neighbor joining phylogenetic tree of 6 *tetA*(48) genes derived from *Pandoraea* sp. XY-2 and other representative species. Bootstrap values are indicated at each node based on a total of 1,000 bootstrap replicates. The genes of *Pandoraea* sp. XY-2 were marked in orange. The green dot and red dot indicated that each strain of *Pandoraea* spp. was isolated from environment and clinical, respectively.

**Figure 11:**
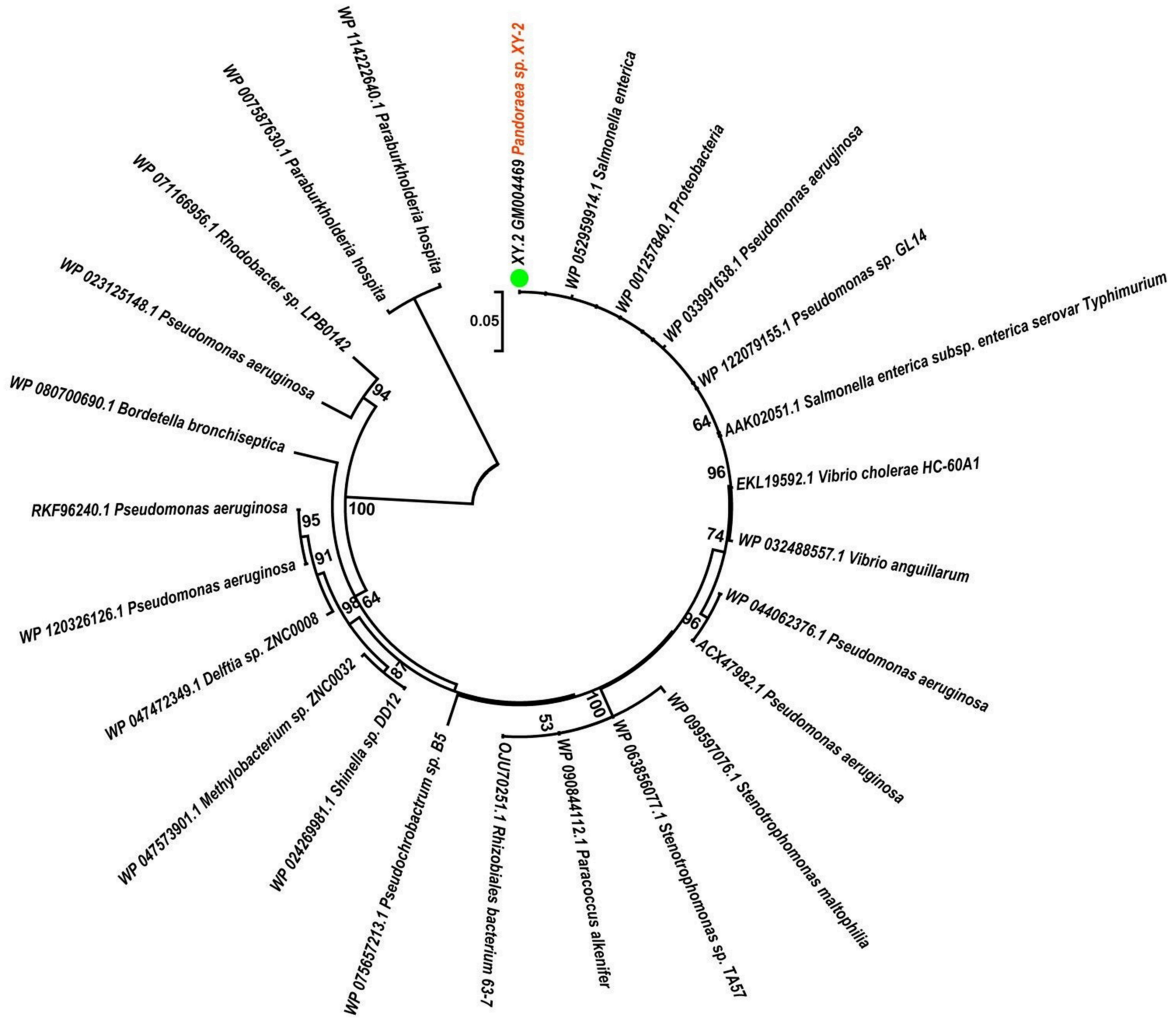
Neighbor joining phylogenetic tree of *tetG* gene derived from *Pandoraea* sp. XY-2 and other representative species. Bootstrap values are indicated at each node based on a total of 1,000 bootstrap replicates. The genes of *Pandoraea* sp. XY-2 were marked in orange. The green dot indicated that *Pandoraea* sp. XY-2 was isolated from environment.

**Figure 12:**
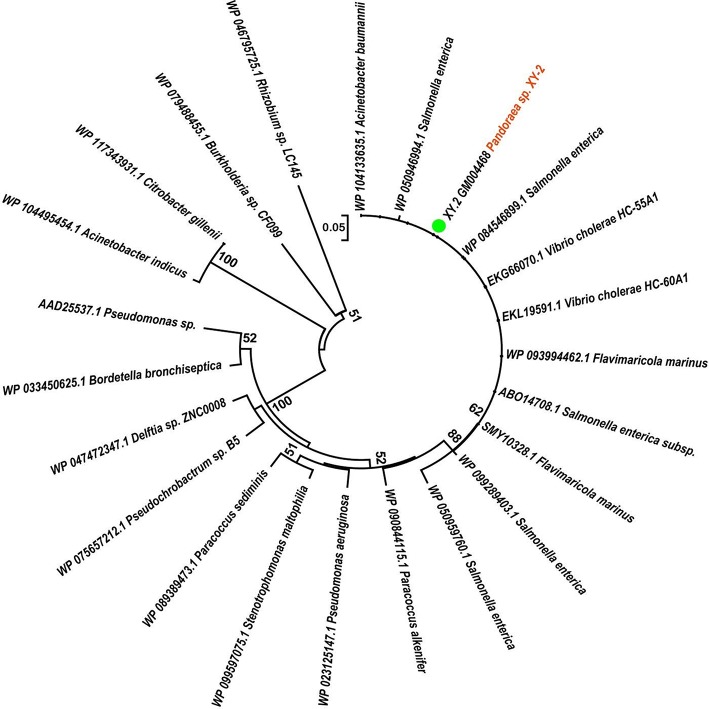
Neighbor joining phylogenetic tree of *tetR* gene derived from *Pandoraea* sp. XY-2 and other representative species. Bootstrap values are indicated at each node based on a total of 1,000 bootstrap replicates. The genes of *Pandoraea* sp. XY-2 were marked in orange. The green dot indicated that *Pandoraea* sp. XY-2 was isolated from environment.

To gain insights into the origin of tetracycline resistance genes in *Pandoraea*, three NJ phylogenetic trees were constructed based on the *tetA*(48), *tetG*, and *tetR* genes encoding protein sequences (Figures 10–12). As shown in Figure 10, phylogenetic analysis revealed that three *tetA*(48) genes (XY.2_GM000586, XY.2_GM000605 and XY.2_GM004567) of *Pandoraea* and tetA(48) genes of *Paraburkholderia* spp., *Burkholderia* spp., and *Caballeronia* spp. were sister groups. It is obviously to note that, the other three *tetA*(48) genes (XY.2_GM001703, XY.2_GM003133 and XY.2_GM003479) of *Pandoraea* sp. XY-2 formed monophyletic clade, respectively. Furthermore, the *tetG* gene from *Pandoraea* sp. XY-2 grouped to *tetG* gene of *S. enterica, V. cholerae, P. aeruginosa*, and *Proteobacteria* (Figure 11). The *tetR* gene from *Pandoraea* sp. XY-2 grouped to *tetR* gene of *S. enterica, V. cholerae, A. baumannii*, and *F. marinus* (Figure 12). These results imply that three *tetA*(48) genes (XY.2_GM000586, XY.2_GM004567, and XY.2_GM000605) may be acquired via HGT from members of *Paraburkholderia, Burkholderia*, and *Caballeronia*. While, the other three *tetA*(48) genes (XY.2_GM001703, XY.2_GM003133, and XY.2_GM003479) may be acquired via HGT from unidentified sources in early evolutionary history. The *tetG* gene may be acquired via HGT from *S. enterica, V. cholerae, P. aeruginosa* and *Proteobacteria*, and *tetR* from *S. enterica, V. cholerae, A. baumannii*, and *F. marinus*.

## Discussion

The data we present represents a first glimpse into the evolution and mechanism for tetracycline biodegradation and resistance in genus *Pandoraea*. Different from sulfonamide compounds, whose degradation by pure bacterial cultures have been reported (Tappe et al., 2013), there are only a few microorganisms that can degrade tetracycline. Previous literature reports that *Stenotrophomonas maltophilia* strain DT1 can effectively biotransform tetracycline, which can degrade about 46% tetracycline at 30°C and pH 9 in 7 days (Leng et al., 2016). Huang et al. (2016) observed that *Trichosporon mycotoxinivorans* XPY-10 which was isolated from wastewater could degrade about 46.28% tetracycline at 34°C within 7 days. Zhang et al. (2015) reports that 57.8% tetracycline could be degraded by *Advenella kashmirensi* at 30°C and pH 7 in 6 days. A recent study showed that a maximum tetracycline degradation ratio of 78.78% can be obtained by *Klebsiella* sp. SQY5 at an initial pH of 7.17, a temperature of 34.96°C, an initial tetracycline concentration of 61.27 mg L^−1^ and an inoculation dose of 29.89% (v/v) using SAS software prediction (Shao et al., 2018). In this study, *Pandoraea* sp. XY-2 was screened out, which could biodegrade 74% tetracycline at pH 7.0 and 30°C within 6 days. At present, the functional characteristics of *Pandoraea* have been reported. Previously identified *Pandoraea* spp. are predominantly clinical isolates with a few strains being associated with biodegradation of environmental pollutants (Lim et al., 2016). For example, *Pandoraea* participates in the bioremediation of lignin, plasticizer dibutyl phthalate, phenol and petroleum hydrocarbon pollutants (Sarkar et al., 2017; Fang et al., 2018; Liu et al., 2018; Yang et al., 2018). In short, it has not yet been reported that tetracycline could be biodegraded by genus *Pandoraea*. Therefore, this is the first discovery that *Pandoraea* sp. XY-2 can efficiently biodegrade tetracycline, and its biodegradation percentage was about 74%, which indicates that *Pandoraea* sp. XY-2 is an excellent microorganism resource for tetracycline wastewater treatment.

Although the cell density of *Pandoraea* sp. XY-2 decreased during decline period, the active cells could continue to biodegrade tetracycline. Therefore, the biodegradation percentage of tetracycline increased slightly as the cell density of *Pandoraea* sp. XY-2 decreased (Figure 1). Similar to the findings from a previous study (Loftin et al., 2008), this study demonstrated that high temperatures accelerated tetracycline hydrolysis. For the temperature tested, the biodegradation percentage of tetracycline does not decrease as biomass decreased drastically from 35 to 40°C (Figure 2). According to the collision theory, higher temperatures lead to higher reaction rates by causing more collisions between particles (Thornton, 1964). Moreover, high pH also affected the removal of tetracycline, when the initial pH was 9, the biomass of *Pandoraea* sp. XY-2 decreased but the tetracycline biodegradation percentage increased (Figure 3). This may be because tetracycline can form invalid lactone isomers easily under alkaline conditions (Hallingsørensen et al., 2003). The pH value of the sampling location in this study was about 6.8, which was not conducive to the natural degradation of tetracycline. The optimum pH for *Pandoraea* sp. XY-2 biodegrading tetracycline is 7.0, so it may play an important role in the *situ* remediation of tetracycline-contaminated environment.

A detailed understanding of the molecular mechanisms that xenobiotic biodegradation and drug-resistance in genus *Pandoraea* is optimally addressed using truly comprehensive information about their genomes. Now, the whole genome of *Pandoraea* sp. XY-2 was successively sequenced using the Pacific Biosciences RS II in the present study. Unsurprisingly, the *Pandoraea* sp. XY-2 exhibited similarities in genome size and GC content compared to the other *Pandoraea* strains (Table 1). Moreover, the phylogenetic analysis confirmed that *Pandoraea* sp. XY-2 was most closely related to the *Pandoraea* sp. 64–18 and *Pandoraea* sp. B-6, implying that *Pandoraea* sp. XY-2, *Pandoraea* sp. 64–18, and *Pandoraea* sp. B-6 have a common ancestor. With the rapid advances in genomics analysis technology, new measurements such as ANI are being developed to evaluate the genomic similarity between bacteria (Richter and Rossellómóra, 2009). Here, the nucleotide comparison of the whole genome sequence of *Pandoraea* sp. XY-2 with other *Pandoraea* species revealed an ANI% value < 85%. An additional, the dDDH% values of *Pandoraea* sp. XY-2 against all reference strains were less 52.2%. Both ANI and dDDH values described herein were considerably below the threshold for species circumscription (Richter and Rossellómóra, 2009). Therefore, we can consider that *Pandoraea* sp. XY-2 belongs to a new species.

The CAZymes annotation of *Pandoraea* sp. XY-2 genome revealed two genes (XY.2_GM002965 and XY.2_GM003795) encoding AA6 family members in its genome sequence. All experimentally characterized proteins in AA6 family were 1,4-benzoquinone reductases, which were intracellular enzymes involved in the biodegradation of aromatic compounds (Spain and Gibson, 1991). It suggested that these enzymes might play an important role in the biodegradation of tetracycline. On the other hand, the COG annotation revealed a large number of genes involved in carbohydrate transport and metabolism, secondary metabolites biosynthesis, transport and catabolism and intracellular trafficking, secretion, and vesicular transport. The existence of these genes may be closely related to the ability of *Pandoraea* sp. XY-2 to biodegrade tetracycline. The GO annotation results indicated that the protein function of *Pandoraea* sp. XY-2 was mainly focused on cell composition, enzyme catalysis and metabolism. Furthermore, KEGG annotation showed that 9 genes encoding dioxygenase. Dioxygenase can degrade aromatic compounds by dihydroxylation, monohydroxylation and tandem oxidation (Boyd et al., 2001). Nine GSTs genes were also identified in the genome sequence of *Pandoraea* sp. XY-2. Previous reports showed that tetracycline can be more readily biodegradable by using the detoxifying enzyme GSTs, and the biotoxicity of tetracycline was greatly reduced (Park and Choung, 2007). The comparative analysis of all the 9 GSTs-encoding genes in *Pandoraea* sp. XY-2 together with GSTs- encoding genes from close-related strains revealed that the GSTs-encoding genes were present in both environmental and clinical isolates and the contents of these genes did not change significantly. It indicated that there may be no major variation in the ability to degrade tetracycline between these isolates irrespective of their origin. Similar conclusion about environmental strains and clinical ones shared similar number of resistance determinants was also reported by Youenou et al. (2015).

As part of the genomic characterization, the pan and core genome of *Pandoraea* sp. XY-2 were defined as a way to cluster existing genes in all analyzed genomes. Our pan-genome analysis of strains in the genus *Pandoraea* revealed that *Pandoraea* sp. XY-2 had 414 specific genes, which could be related to the unique characteristics of *Pandoraea* sp. XY-2. As expected, the KEGG annotation of the specific genes in *Pandoraea* sp. XY-2 revealed 2 important genes *tetG* and *tetR* related to tetracycline resistance. The most important example is the *tetR* gene, which encoding multi-drug binding proteins AcrR. According to previous reports, the multi-drug binding proteins AcrR is member of the TetR family of transcriptional repressors that regulate the expression of the multidrug resistant efflux pumps AcrAB (Routh et al., 2009). In short, these results provided some clues on the specific genes involved in tetracycline resistance development and molecular basis of tetracycline biodegradation capacity in the studied strain of *Pandoraea* sp. XY-2. On the other hand, the model extrapolation revealed that the number of core genes was relatively stable that an extra genome added would not significantly affect its size, which was consistent with the notion that core genes were conserved genes universally present in all strains (Zhang and Sievert, 2014). Besides, comparison of the complete sequence of the *Pandoraea* sp. XY-2 and *P. apista* DSM16535^T^ revealed a total of 67,476 non-synonymous SNPs and 581 InDels were detected in the genome of *Pandoraea* sp. XY-2. The SNPs, InDels and different syntenial relationships identified here might be associated with differences in biodegradation characteristics of organic contaminants and drug resistance of *Pandoraea* spp., and also contribute to understanding the phylogeny and population genetics of *Pandoraea* spp.

In order to providing insights into the molecular basis of tetracycline biodegradation by *Pandoraea* sp. XY-2, we identified the antibiotic resistance genes by CARD analysis. The results showed that *Pandoraea* sp. XY-2 was resistant to various antibiotics, suggesting multiple resistance mechanisms presented in *Pandoraea* sp. XY-2. Particularly, we also identified the efflux pump-encoding genes *tetA*(48) and *tetG* and the ribosome protection protein-encoding gene *tetB*(P) and *tetT*, which was associated with tetracycline resistance. The efflux pump system can pump tetracycline molecules out of a cell and ribosomal protection proteins can cause an allosteric disruption on tetracycline binding sites and release tetracycline from the ribosome (Thaker et al., 2010; Roberts et al., 2011). Furthermore, efflux pumps play a major role in both solvent tolerance and bioremediation (Fernandes et al., 2003). Therefore, the existence of these resistance genes indicated that the *Pandoraea* sp. XY-2 had the molecular basis of tetracycline resistance, which makes it possible and precondition to biodegrade tetracycline.

Horizontal gene transfer (HGT) mediated by plasmids, transposons, or phages is considered to be the mechanism responsible for the widespread distribution of biodegradative pathways in bacteria (De and Davies, 2000). The deviant G+C content is one of the indicative used to detect HGT (Xie et al., 2014). We implemented a combination of deviant G+C content detection and phylogenetic analysis to discover putative HGT events since it is difficult to identify HGT events via deviant G+C content alone if HGT happened to occur between the organisms with the similar G+C contents (Hirsch et al., 1995). The different gene content and organization of antibiotic resistance genes suggest complicated evolutionary history of these genes, and also suggest different genetic requirements for effective cellular antibiotic detoxification and regulation of antibiotic resistance operons. Our results supposed that the *tetA*(48), *tetG*, and *tetR* genes in *Pandoraea* spp. may be acquired by HGT from *Paraburkholderia* spp., *Burkholderia* spp., *Caballeronia* spp., *S. enterica, V. cholerae, Proteobacteria, P. aeruginosa, A. baumannii, F. marinus*, and some unidentified sources in early evolutionary history. Similar conclusion about gram-positive and gram-negative bacteria tetracycline resistance was a trait acquired via HGT was also reported by Mindlin et al. (2006). Previous studies have shown that *Burkholderia cepacia*, with genetic determinants for efflux pumps that facilitate tetracycline excretion, was the only bacterium that grew on tetracycline-amended R2A plates (Rysz and Alvarez, 2004). Moreover, Todorovic et al.' 2015 have detected the *tetA* gene and the corresponding *tetR* gene in the *Salmonella* Infantis isolated from 28 poultry farms in the Northern part of Serbia. The determination of tetracycline resistance genes in *V. cholerae* have also been researched by Khany et al. (2016).

## Conclusions

*Pandoraea* sp. XY-2 has good performance for biodegrade tetracycline, which could biodegrade 74% tetracycline at pH 7.0 and 30°C within 6 days. Genomic analyses including ANI and dDDH provided a clear result that the *Pandoraea* sp. XY-2 belongs to a new species. Additionally, the analysis of genome characteristics and comparative genome revealed that *Pandoraea* sp. XY-2 contained AA6 family members-encoding genes, GSTs-encoding genes and some tetracycline resistance genes, such as *tetA*(48), *tetB*(P), *tet*T, *tetG*, and *tetR*, which were relevant to tetracycline biodegradation and resistance. In the near future, the transcriptomics and proteomic technology will be conducted to further explore the molecular mechanism of tetracycline biodegradation by *Pandoraea* sp. XY-2.

## Data Availability

The sequence data of the whole genome of *Pandoraea* sp. XY-2 have been deposited to NCBI data base, with the accession number of CP030849.

## Author Contributions

XuW, XiW, and WZ designed and coordinated the study and carried out the data analysis. XuW, XiW, LS, and WZ performed the bioinformatics analysis. XuW, XiW, JL, and RY carried out the experiments and interpreted data for the work. XuW, XiW, and WZ wrote the manuscript. GQ checked and edited the manuscript. All authors have read and approved the manuscript.

### Conflict of Interest Statement

The authors declare that the research was conducted in the absence of any commercial or financial relationships that could be construed as a potential conflict of interest.
