# Supplementary material for: Whole Genome Sequencing and Comparative Genomics Analyses of Pandoraea sp. XY-2, a New Species Capable of Biodegrade Tetracycline
Source: Front Microbiol. 2019 Jan 29;10:33. doi: 10.3389/fmicb.2019.00033 (PMC6361800; doi:10.3389/fmicb.2019.00033)
Supplement: Supplementary file 1 [file Data_Sheet_1.docx]

***Supplementary Material***

**Whole genome sequencing and comparative genomics analyses of *Pandoraea* sp. XY-2, a new species capable of biodegrade tetracycline**

**Xueling Wu^1,2^, Xiaoyan Wu^1^, Li Shen^1,2^, Jiaokun Li^1,2^, Runlan Yu^1,2^, Yuandong Liu^1,2^, Guanzhou Qiu^1,2^ and Weimin Zeng^1,2*^**

*** Correspondence:** Weimin Zeng: [zengweimin1024@126.com](mailto:zengweimin1024@126.com)

**Supplementary Data**

As shown in Supplementary Figure 1, in blank group, the removal of tetracycline was processed by hydrolysis, the removal percentage of tetracycline increased slowly, reached about 5% in 108 h, and then keep it stable. However, the removal percentage of tetracycline normal group reached74.87% in 144h. So, the hydrolysis of tetracycline could be ignored, only biodegradation was considered in this study.

**Supplementary Figure legends**

**Supplementary Figure 1.** Changes of tetracycline removal percentage over time under various treatment conditions. The initial concentration of tetracycline was 50 mg L^-1^. TC: tetracycline. In normal group, *Pandoraea* sp. XY-2 were cultured in 150 mL LB medium; In blank group, only 150 mL medium was used (without *Pandoraea* sp. XY-2).

**Supplementary Figure 2. Scanning electron microscope image of the *Pandoraea* sp. XY-2.**

**Supplementary Figure 3.** Six classification statistics of CAZy database.

**Supplementary Figure 4.** COG distribution of the *Pandoraea* sp. XY-2 genome annotation.

**Supplementary Figure 5.** GO classification statistics of the *Pandoraea* sp. XY-2 genome annotation.

**Supplementary Figure 6.** KEGG classification statistics of the *Pandoraea* sp. XY-2 genome annotation.

**Supplementary Figure 7.** Neighbor joining phylogenetic tree of 9 GSTs-encoding genes derived from *Pandoraea* sp. XY-2 and other close-related strains. Bootstrap values are indicated at each node based on a total of 1,000 bootstrap replicates. *Pandoraea* sp. XY-2 were marked in orange. The green dot and red dot indicated that each strain of *Pandoraea* spp. was isolated from environment and clinical, respectively.

**Supplementary Figure 8.** Neighbor-joining (NJ) phylogenetic tree of *Pandoraea* sp. XY-2 based on 16S rRNA gene sequence. The other reference sequences were retrieved from the EzTaxon server. Bootstrap values are indicated at each node based on a total of 1,000 bootstrap replicates.

**Supplementary Figure 9.** KEGG classification statistics of the specific genes of *Pandoraea* sp. XY-2.

**Supplementary Figure 10.** Phylogenetic analysis of *Pandoraea* sp. XY-2 based on all of the single-copy core genes among 19 whole genome sequences. The phylogeny tree was generated using the NJ method in Mega 7.0 with 1000 bootstrap replicates. The whole genome sequences of 18 other *Pandoraea* strains, which genome sequences level were complete, were retrieved from GenBank.

**Supplementary Figures**

**

**

**Supplementary Figure 1.**

**
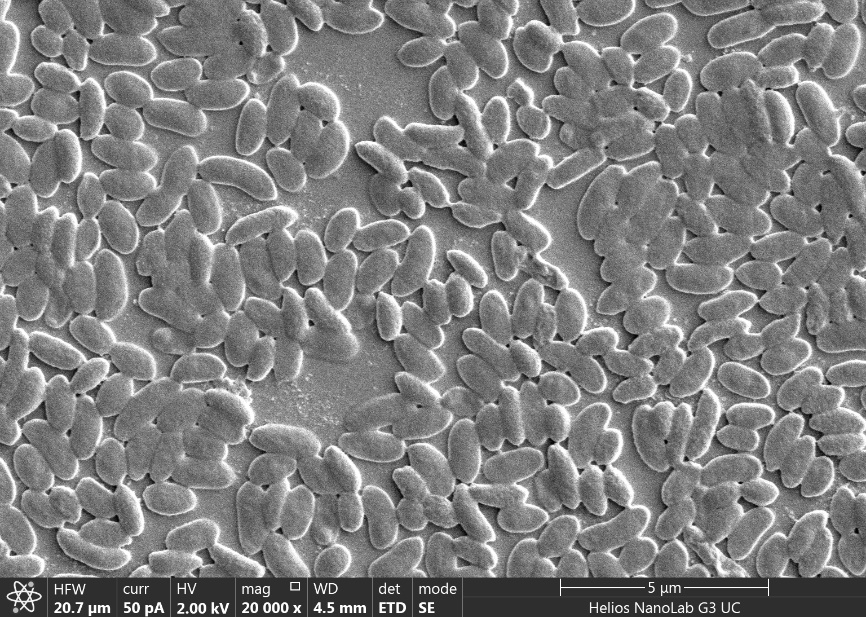
**

**Supplementary Figure 2.**

**
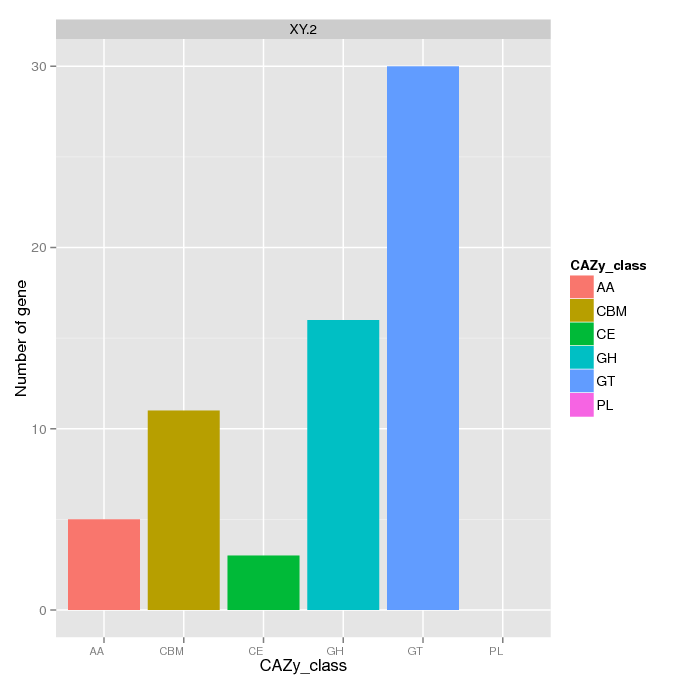
**

**Supplementary Figure 3.**

**
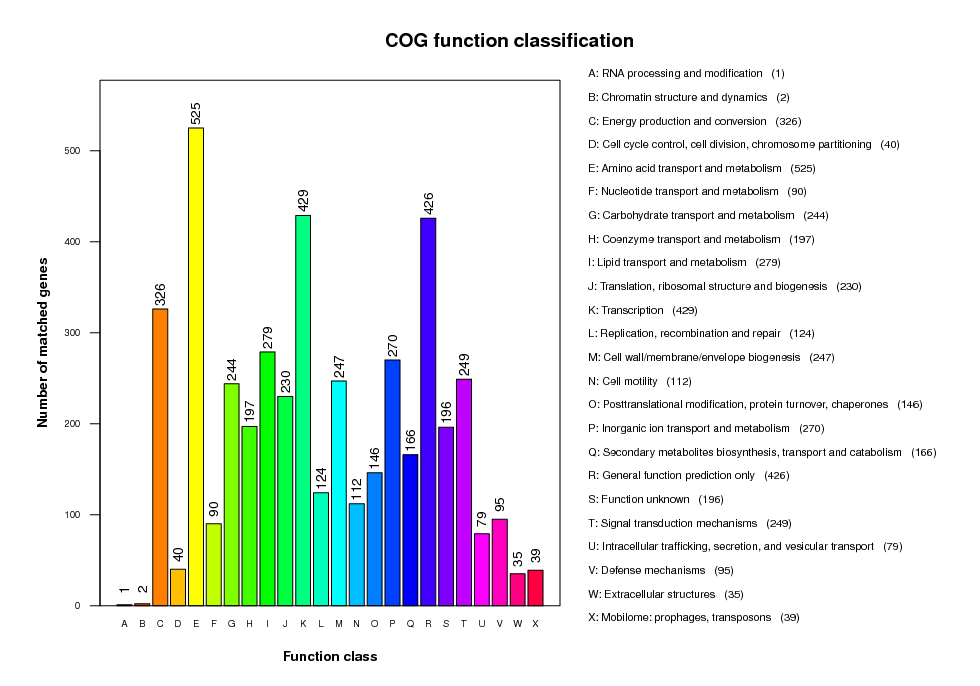
**

**Supplementary Figure 4.**

**
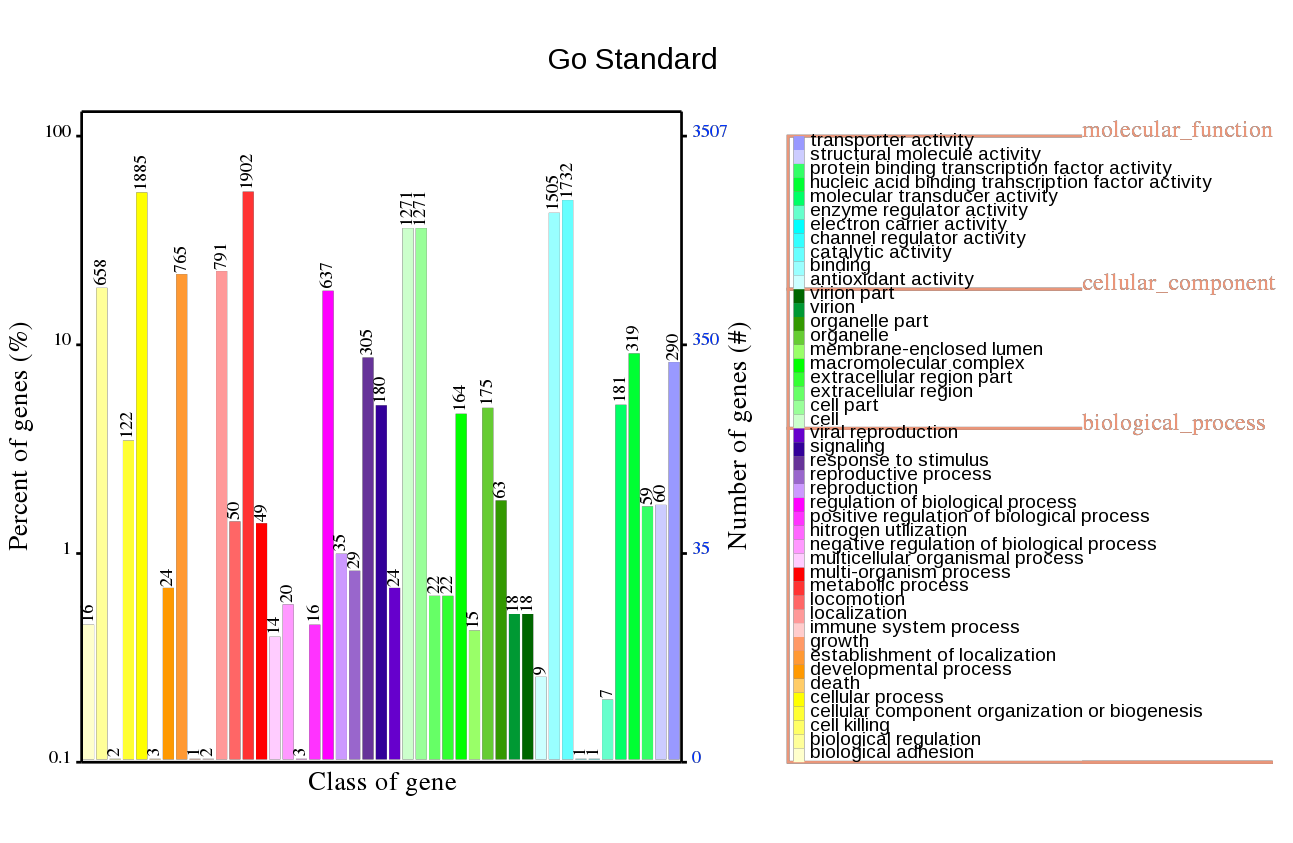
**

**Supplementary Figure 5.**

**
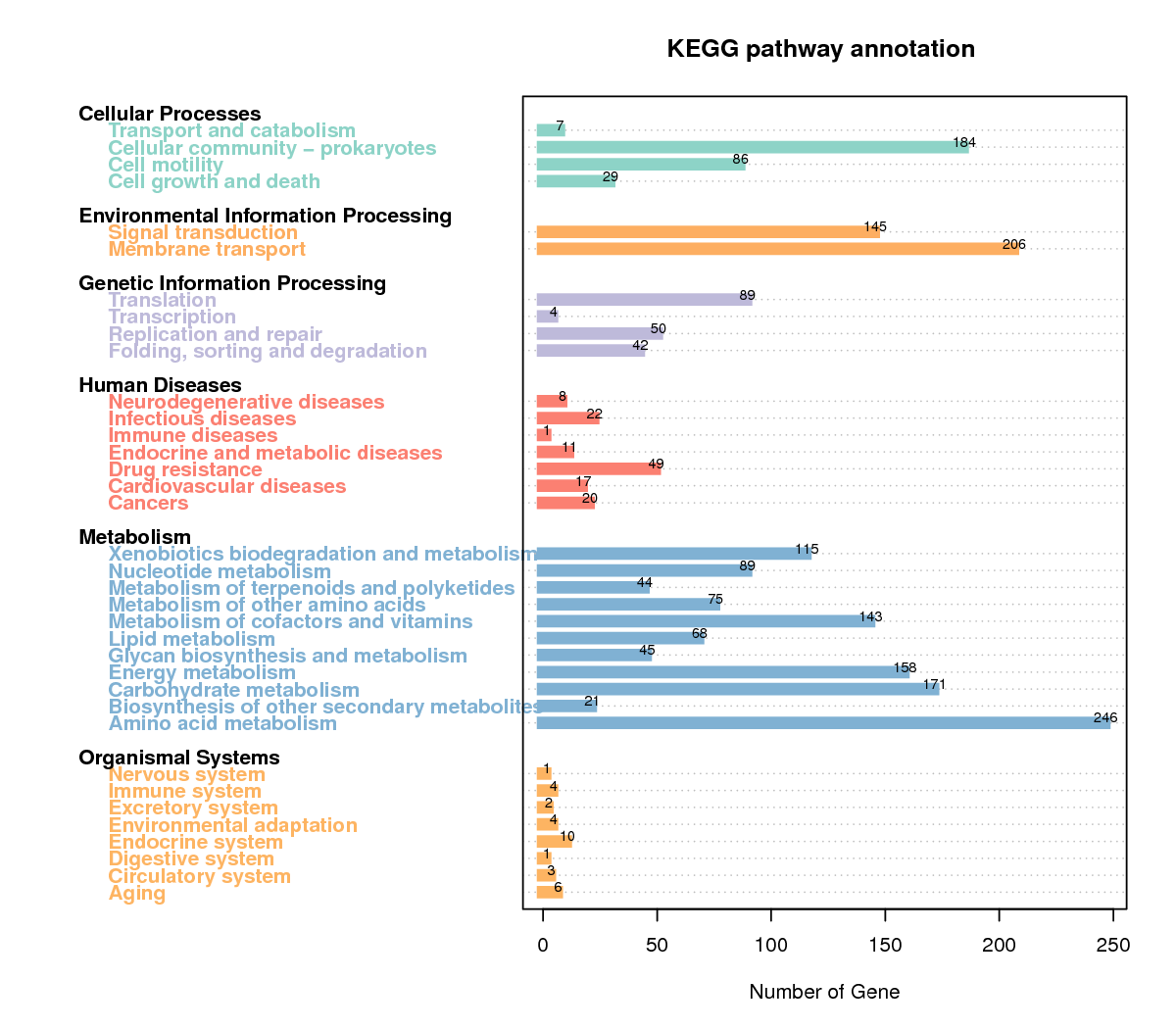
**

**Supplementary Figure 6.**

**
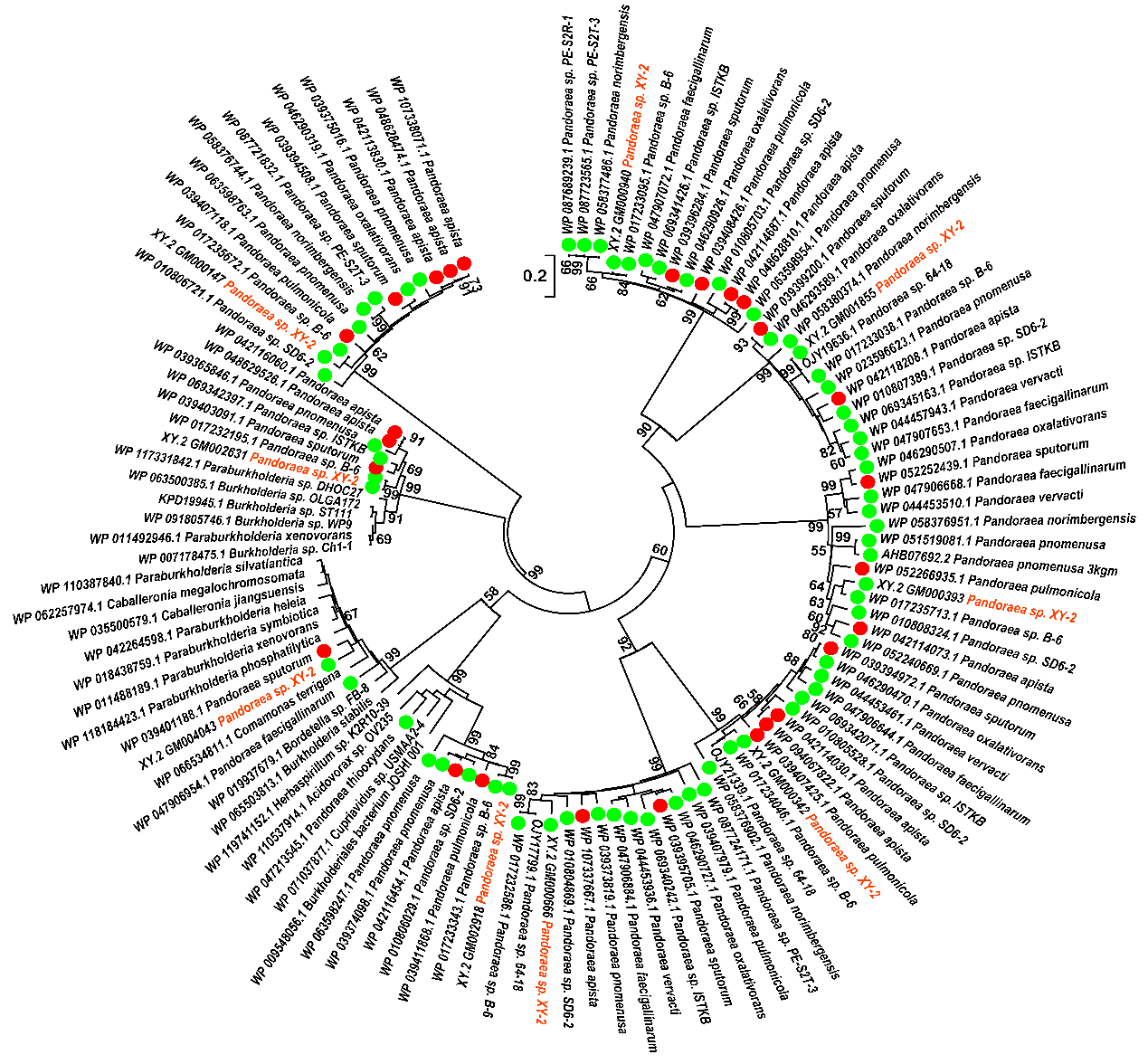
**

**Supplementary Figure 7**

**
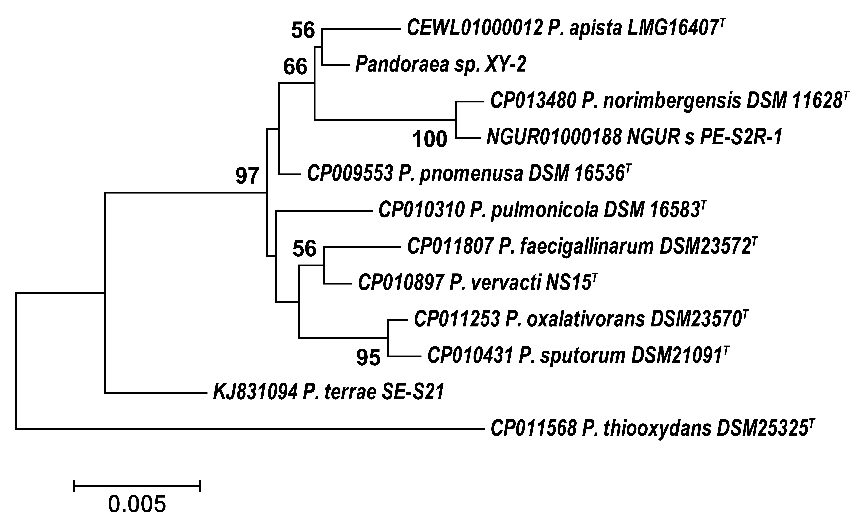
**

**Supplementary Figure 8**

**

**

**Supplementary Figure 9.**

**
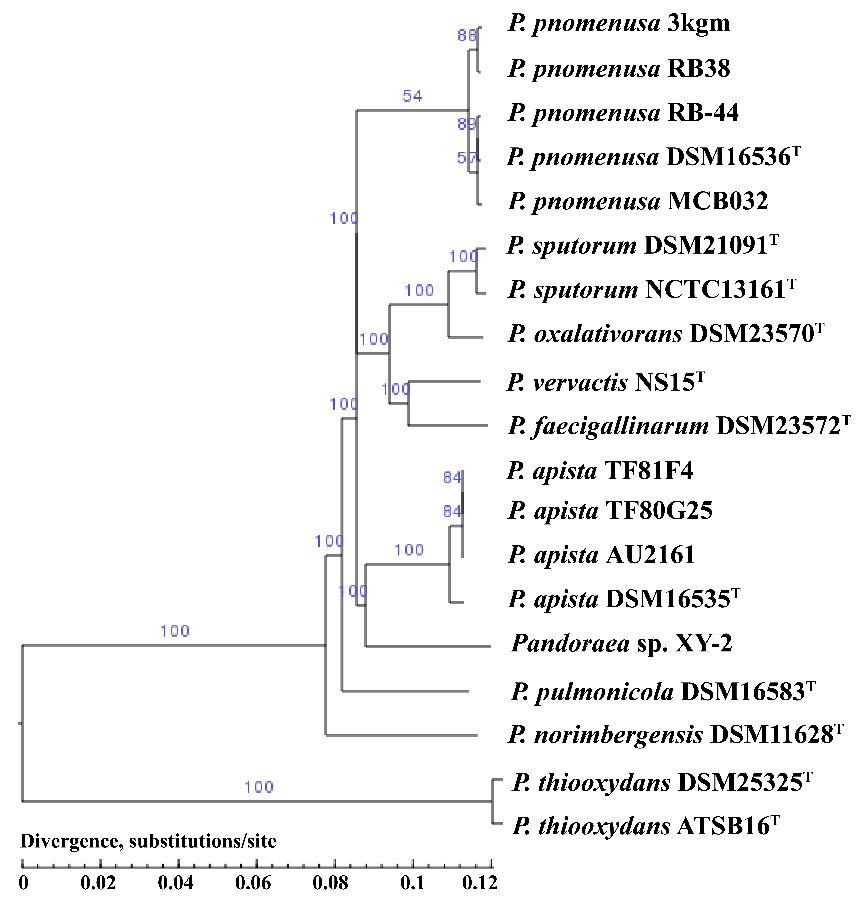
**

**Supplementary Figure 10.**

**Supplementary Tables**

**Supplementary Table S1 The details source and the GSTs-encoding gene content of all testing strains.**

| Organism | Source | Number of GSTs-encoding gene |
| --- | --- | --- |
| *P. pnomenusa* DSM 16536^T^ | CF patient (Lim et al., 2016) | 13 |
| *P. pnomenusa* RB-44 | exlandfill dumping ground (Ee et al., 2014) | 12 |
| *P. pnomenusa* 3kgm | former landfill site (Chan et al., 2013) | 13 |
| *P. pnomenusa* RB38 | municipal solid waste landfill site (Lim et al., 2015) | 13 |
| *P. pnomenusa* MCB032 | functionally stable bioreactor (Baptista et al., 2010) | 13 |
| *P. pnomenusa* 6399 | CF patient^b^ | 14 |
| *P. pnomenusa* 7641 | CF patient^b^ | 14 |
| *P. pnomenusa* NCTC13160 | unkown | 13 |
| *P. pnomenusa* RB44 | soil^b^ | 12 |
| *P. norimbergensis* DSM11628^T^ | food (Moore, 2001) | 13 |
| *P. faecigallinarum* DSM23572^T^ | oxalate-enriched culture (Nurettin et al., 2000) | 10 |
| *P. thiooxydans* ATSB16^T^ | rhizosphere soils of sesame (Rangasamy et al., 2010) | 15 |
| *P. thiooxydans* DSM25325 ^T^ | rhizosphere soils of sesame (Yong et al., 2016) | 15 |
| *P. oxalativorans* DSM23570^T^ | soil (Chan et al., 2016) | 9 |
| *P.* *apista* TF81F4 | sputum^b^ | 14 |
| *P. apista* TF80G25 | sputum^b^ | 14 |
| *P. apista* AU2161 | sputum^b^ | 14 |
| *P.apista* FDAARGOS_126 | sputum^b^ | 14 |
| *P. apista* LMG16407^T^ | CF patient (Ackiss et al., 2011) | 14 |
| *P. apista* Pa14367 | sputum^b^ | 14 |
| *P. apista* Pa16226 | sputum^b^ | 14 |
| *P. apista* DSM16535 ^T^ | CF patient (Ackiss et al., 2011) | 14 |
| *P. vervacti* NS15^T^ | uncultivated field soil (Ee et al., 2015) | 12 |
| *P. pulmonicola* DSM 16583^T^ | CF patient (Coenye et al., 2000) | 14 |
| *P. pulmonicola* NCTC13159 | CF patient (Coenye et al., 2000) | 14 |
| *P. sputorum* DSM21091 ^T^ | CF patient (Coenye et al., 2000) | 12 |
| *P. sputorum* NCTC13161 ^T^ | CF patient (Coenye et al., 2000) | 12 |
| *Pandoraea* sp. 64-18 | unkown | 13 |
| *Pandoraea* sp. B-6 | bamboo slips (Shi et al., 2013) | 8 |
| *Pandoraea* sp. CB10b_02 | unkown | unkown |
| *Pandoraea* sp. E26 | former landfill site (Chan et al., 2015) | 12 |
| *Pandoraea* sp. ISTKB | rhizospheric zone of trees (Kumar et al., 2016) | 14 |
| *Pandoraea* sp. PE-S2R-1 | forest^b^ | 15 |
| *Pandoraea* sp. PE-S2T-3 | forest^b^ | 15 |
| *Pandoraea* sp. SD6-2 | lindane-contaminated soil (Hafizah et al., 2013) | 12 |
| *Pandoraea* sp. XY-2^a^ | tetracycline-contaminated sludge | 9 |

**^a^ This study. ^b^ represents informations obtained from the EzBioCloud Database (https://www.ezbiocloud.net/).**

**Supplementary Table S3 16SrRNA gene sequence similarity of *Pandoraea* sp. XY-2 and the type strains of *Pandoraea* spp. using EzTaxon server.**

| Organism | 16SrRNA accession | Similarity (100%) |
| --- | --- | --- |
| *P. pnomenusa* DSM 16536^T^ | CP009553 | 99.72 |
| *P. norimbergensis* DSM 11628^T^ | CP013480 | 99.17 |
| *P. faecigallinarum* DSM23572^T^ | CP011807 | 98.97 |
| *P. thiooxydans* DSM25325^T^ | CP011568 | 96.9 |
| *P. oxalativorans* DSM23570^T^ | CP011253 | 99.24 |
| *P. apista* LMG16407^T^ | CEWL01000012 | 99.59 |
| *P. vervacti* NS15^T^ | CP010897 | 99.24 |
| *P. pulmonicola* DSM 16583^T^ | CP010310 | 99.24 |
| *P. sputorum* DSM21091^T^ | CP010431 | 99.04 |
| *P. terrae* SE-S21 | KJ831094 | 98.69 |
| *NGUR_s* PE-S2R-1 | NGUR01000188 | 99.24 |

**Supplementary Table S4 Digital DNA-DNA hybridization (dDDH) between *Pandoraea* genomes.**

|  |  | | | | Formula 1 | | |  | | | Formula 2 | |  | |  | | | Formula 3 | |  | |
| --- | --- | --- | --- | --- | --- | --- | --- | --- | --- | --- | --- | --- | --- | --- | --- | --- | --- | --- | --- | --- | --- |
| Query genome | | Reference genome | DDH | Model C.I. | | Distance | Prob. DDH >= 70% | | DDH | Model C.I. | | Distance | | Prob. DDH >= 70% | | DDH | Model C.I. | | Distance | | Prob. DDH >= 70% |
| XY-2 | | CP009553 | 44.5 | [41.1 - 47.9%] | | 0.351 | 4.53 | | 27.5 | [25.1 - 30%] | | 0.1569 | | 0.03 | | 39.2 | [36.2 - 42.2%] | | 0.4529 | | 0.1 |
| XY-2 | | CP006938 | 44.5 | [41.1 - 48%] | | 0.3507 | 4.57 | | 27.5 | [25.2 - 30%] | | 0.1567 | | 0.03 | | 39.2 | [36.3 - 42.3%] | | 0.4524 | | 0.1 |
| XY-2 | | CP006900 | 44.1 | [40.7 - 47.5%] | | 0.3543 | 4.19 | | 27.5 | [25.1 - 30%] | | 0.1569 | | 0.03 | | 38.9 | [36 - 42%] | | 0.4557 | | 0.09 |
| XY-2 | | CP007506 | 44.8 | [41.4 - 48.2%] | | 0.3481 | 4.85 | | 27.5 | [25.2 - 30%] | | 0.1568 | | 0.03 | | 39.4 | [36.5 - 42.5%] | | 0.4503 | | 0.11 |
| XY-2 | | CP015371 | 43.4 | [40.1 - 46.9%] | | 0.3602 | 3.66 | | 27.5 | [25.2 - 30%] | | 0.1567 | | 0.03 | | 38.5 | [35.5 - 41.5%] | | 0.4604 | | 0.08 |
| XY-2 | | CP013480 | 33.2 | [29.8 - 36.8%] | | 0.4687 | 0.26 | | 26.2 | [23.9 - 28.7%] | | 0.1654 | | 0.02 | | 30.5 | [27.6 - 33.6%] | | 0.5566 | | 0 |
| XY-2 | | CP011807 | 40 | [36.6 - 43.5%] | | 0.3924 | 1.69 | | 27.2 | [24.8 - 29.6%] | | 0.1591 | | 0.03 | | 35.8 | [32.9 - 38.9%] | | 0.489 | | 0.03 |
| XY-2 | | CP014839 | 14.9 | [12 - 18.3%] | | 0.8834 | 0 | | 20.7 | [18.5 - 23.1%] | | 0.2122 | | 0 | | 15 | [12.6 - 17.9%] | | 0.9081 | | 0 |
| XY-2 | | CP011568 | 14.9 | [12 - 18.3%] | | 0.8834 | 0 | | 20.7 | [18.5 - 23.1%] | | 0.2124 | | 0 | | 15 | [12.6 - 17.9%] | | 0.9082 | | 0 |
| XY-2 | | CP011253 | 39.8 | [36.4 - 43.2%] | | 0.3949 | 1.59 | | 27.3 | [24.9 - 29.7%] | | 0.1585 | | 0.03 | | 35.7 | [32.7 - 38.8%] | | 0.4908 | | 0.03 |
| XY-2 | | CP010518 | 50 | [46.6 - 53.5%] | | 0.3067 | 12.35 | | 28.8 | [26.4 - 31.3%] | | 0.149 | | 0.06 | | 43.7 | [40.7 - 46.7%] | | 0.41 | | 0.46 |
| XY-2 | | CP011279 | 50 | [46.6 - 53.5%] | | 0.3065 | 12.39 | | 28.8 | [26.4 - 31.3%] | | 0.149 | | 0.06 | | 43.7 | [40.7 - 46.7%] | | 0.4098 | | 0.46 |
| XY-2 | | CP011501 | 49.8 | [46.4 - 53.3%] | | 0.308 | 12 | | 28.8 | [26.4 - 31.3%] | | 0.1489 | | 0.06 | | 43.6 | [40.6 - 46.6%] | | 0.411 | | 0.44 |
| XY-2 | | CP013481 | 49.4 | [45.9 - 52.8%] | | 0.3117 | 11.08 | | 28.9 | [26.5 - 31.4%] | | 0.1483 | | 0.07 | | 43.3 | [40.3 - 46.3%] | | 0.4138 | | 0.4 |
| XY-2 | | CP014018 | 52.2 | [48.8 - 55.7%] | | 0.2906 | 17.28 | | 28.7 | [26.4 - 31.2%] | | 0.1492 | | 0.06 | | 45.2 | [42.2 - 48.2%] | | 0.3965 | | 0.73 |
| XY-2 | | CP010897 | 36.8 | [33.4 - 40.3%] | | 0.4259 | 0.75 | | 27.2 | [24.9 - 29.7%] | | 0.1586 | | 0.03 | | 33.5 | [30.6 - 36.6%] | | 0.517 | | 0.01 |
| XY-2 | | CP010310 | 38.3 | [34.9 - 41.7%] | | 0.4102 | 1.1 | | 27.8 | [25.4 - 30.2%] | | 0.1553 | | 0.04 | | 34.8 | [31.8 - 37.8%] | | 0.5018 | | 0.02 |
| XY-2 | | CP010431 | 40 | [36.6 - 43.5%] | | 0.3924 | 1.69 | | 27.1 | [24.7 - 29.6%] | | 0.1595 | | 0.03 | | 35.8 | [32.9 - 38.9%] | | 0.4894 | | 0.03 |
| XY-2 | | LT906435 | 40 | [36.6 - 43.5%] | | 0.3925 | 1.69 | | 27.1 | [24.7 - 29.6%] | | 0.1595 | | 0.03 | | 35.8 | [32.9 - 38.9%] | | 0.4894 | | 0.03 |

**Figures in gray denote the dDDH (%) values of *Pandoraea* sp. XY-2 against all reference strains.**

**Supplementary Table S5 Statistic information of the single nucleotide polymorphism (SNPs) of *Pandoraea* sp. XY-2 compare with *P. apista* DSM16535^T^.**

| Type | Number | Percentage (%) |
| --- | --- | --- |
| Total SNPs | 372,574 | 100 |
| Synonymous | 264,974 | 71.12 |
| Nonsynonymous | 67,476 | 18.11 |
| Nonsense mutation | 395 | 0.11 |
| SNPs in CDS | 333,149 | 89.42 |
| SNPs in intergenic | 39,425 | 10.58 |

**Supplementary Table S6 Statistic information of the insertion/deletion variations (InDels) of *Pandoraea* sp. XY-2 compare with *P. apista* DSM16535 ^T^.**

| Type | Number | Percentage (%) |
| --- | --- | --- |
| Total InDels | 581 | 100 |
| Insertion | 228 | 39.24 |
| Deletion | 353 | 60.76 |
| InDels in CDS | 193 | 33.22 |
| InDels in intergenic | 388 | 66.78 |

**Supplementary Table** **S8 Statistics information of tetracycline resistance genes identified in *Pandoraea* sp. XY-2.**

| Gene type | Gene ID | Gene locus |
| --- | --- | --- |
| *TetA*(48) | XY.2_GM000586 | locus=Chr1:592121:593143:- |
| *TetA*(48) | XY.2_GM000605 | locus=Chr1:608628:609431:- |
| *TetA*(48) | XY.2_GM001703 | locus=Chr1:1727464:1728267:+ |
| *TetA*(48) | XY.2_GM003133 | locus=Chr1:3180686:3181561:- |
| *TetA*(48) | XY.2_GM003479 | locus=Chr1:3564880:3565809:+ |
| *TetA*(48) | XY.2_GM004567 | locus=Chr1:462922:4630178:- |
| *TetB*(P) | XY.2_GM002024 | locus=Chr1:2046568:2048664:- |
| *TetG* | XY.2_GM004469 | locus=Chr1:4540924:4542099:+ |
| *TetT* | XY.2_GM004640 | locus=Chr1:4692983:4695085:- |

**REFERENCES**

Ackiss, E., Guzman-Cottrill, J., Wall, M., and Lipuma, J. (2011). An Investigation of Genotypically Clonal *Pandoraea apista* Isolates at a Cystic Fibrosis Center. *Ajic American Journal of Infection Control* 39, E122-E123. doi: 10.1016/j.ajic.2011.04.214

Baptista, I.I.R., Zhou, N.Y., Emanuelsson, E.A.C., Peeva, L.G., Mantalaris, A., and Livingston, A.G. (2010). Evidence of species succession during chlorobenzene biodegradation. *Biotechnology & Bioengineering* 99, 68-74. doi: 10.1002/bit.21576

Chan, K.G., Yin, W.F., Tee, K.K., Chang, C.Y., and Priya, K. (2015). *Pandoraea* sp. Strain E26: Discovery of Its Quorum-Sensing Properties via Whole-Genome Sequence Analysis. *Genome Announc* 3. doi: 10.1128/genomeA.00565-15

Chan, K.G., Yin, W.F., and Goh, S.Y. (2013). Complete Genome Sequence of *Pandoraea pnomenusa* 3kgm, a Quorum-Sensing Strain Isolated from a Former Landfill Site. *Genome Announcements* 2. doi: 10.1128/genomeA.00427-14

Chan, K.G., Yong, D., Ee, R., Lim, Y.L., Yu, C.Y., Tee, K.K., et al. (2016). Complete genome sequence of *Pandoraea oxalativorans* DSM 23570 T, an oxalate metabolizing soil bacterium. *J. Biotechnol.* 219, 124-125. doi: 10.1016/j.jbiotec.2015.12.037

Coenye, T., Falsen, E., Hoste, B., Ohlen, M., Goris, J., Govan, J.R.W., et al. (2000). Description of Pandoraea gen. nov. with *Pandoraea apista* sp. nov., *Pandoraea pulmonicola* sp. nov., *Pandoraea pnomenusa* sp. nov., *Pandoraea sputorum* sp. nov. and *Pandoraea norimbergensis* comb. nov. *International Journal of Systematic & Evolutionary Microbiology* 50 Pt 2, 887. doi: 10.1099/00207713-50-2-887

Ee, R., Lim, Y.L., Yin, W.F., and Chan, K.G. (2014). De Novo Assembly of the Quorum-Sensing *Pandoraea* sp. Strain RB-44 Complete Genome Sequence Using PacBio Single-Molecule Real-Time Sequencing Technology. *Genome Announc* 2. doi: 10.1128/genomeA.00245-14

Ee, R., Yong, D., Yan, L.L., Yin, W.F., and Chan, K.G. (2015). Complete genome sequence of oxalate-degrading bacterium *Pandoraea vervacti* DSM 23571 T. *J. Biotechnol.* 204, 5-6. doi: 10.1016/j.jbiotec.2015.03.020

Hafizah, P., Pearce, S.L., Oakeshott, J.G., Russell, R.J., and Gunjan, P. (2013). Draft Genome Sequence of *Pandoraea* sp. Strain SD6-2, Isolated from Lindane-Contaminated Australian Soil. *Genome Announcements* 1, 340-344. doi: 10.1128/genomea.00415-13

Kumar, M., Singhal, A., and Thakur, I.S. (2016). Comparison of submerged and solid state pretreatment of sugarcane bagasse by *Pandoraea* sp. ISTKB: Enzymatic and structural analysis. *Bioresource Technol.* 203, 18-25. doi: 10.1016/j.biortech.2015.12.034

Lim, Y.L., Ee, R., Yong, D., Tee, K.K., Yin, W.F., and Chan, K.G. (2015). Complete genome of *Pandoraea pnomenusa* RB-38, an oxalotrophic bacterium isolated from municipal solid waste landfill site. *J. Biotechnol.* 214, 83-84. doi: 10.1016/j.jbiotec.2015.09.018

Lim, Y.L., Ee, R., Yong, D., Yu, C.Y., Ang, G.Y., Tee, K.K., et al. (2016). Complete Genome Sequence Analysis of *Pandoraea pnomenusa* Type Strain DSM 16536T Isolated from a Cystic Fibrosis Patient. *Front. Microbiol.* 7, 109. doi: 10.3389/fmicb.2016.00109

Moore, J.E. (2001). First report of *Pandoraea norimbergensis* isolated from food--potential clinical significance. *Food Microbiol.* 18, 113-114. doi: 10.1006/fmic.2000.0375

Nurettin, S., Akio, T., Recep, K., Ivo, S., Kazuhide, K., and Abdurrahman, U.T. (2000). *Pandoraea* *oxalativorans* sp. nov., *Pandoraea faecigallinarum* sp. nov. and *Pandoraea vervacti* sp. nov., isolated from oxalate-enriched culture. International Journal of Systematic and Evolutionary Microbiology, 50, 887–899. doi: 10.1099/ijs.0.026138-0

Rangasamy, A., Pandiyan, I., Soon Wo, K., Min, S.T., Ok, J.C., Ki, K.Y., et al. (2010). *Pandoraea thiooxydans* sp. nov., a facultatively chemolithotrophic, thiosulfate-oxidizing bacterium isolated from rhizosphere soils of sesame (Sesamum indicum L.). *International Journal of Systematic & Evolutionary Microbiology* 60, 21. doi: 10.1099/ijs.0.012823-0

Shi, Y., Chai, L., Tang, C., Yang, Z., Zheng, Y., Chen, Y., et al. (2013). Biochemical investigation of kraft lignin degradation by *Pandoraea* sp. B-6 isolated from bamboo slips. *Bioprocess & Biosystems Engineering* 36, 1957-1965. doi: 10.1007/s00449-013-0972-9

Yong, D., Ee, R., Lim, Y.L., Yu, C.Y., Ang, G.Y., How, K.Y., et al. (2016). Complete genome sequence of *Pandoraea thiooxydans* DSM 25325 T, a thiosulfate-oxidizing bacterium. *J. Biotechnol.* 217, 51-52. doi: 10.1016/j.jbiotec.2015.12.037
